# Inapparent virus infections differentially affect honey bee flight

**DOI:** 10.1126/sciadv.adw8382

**Published:** 2025-08-08

**Authors:** Naomi G. Kaku, Mark A. Jankauski, Bridget F. Doyle, Conrad J. Collins, Michelle L. Flenniken

**Affiliations:** ^1^ Department of Plant Sciences and Plant Pathology, Montana State University, Bozeman, MT, USA.; ^2^ Pollinator Health Center, Montana State University, Bozeman, MT, USA.; ^3^ Department of Mechanical Engineering, Montana State University, Bozeman, MT, USA.

## Abstract

Honey bee colony deaths are associated with viruses, which frequently do not cause morphological symptoms in adult bees. To assess the impact of these inapparent infections, we measured flight performance as a proxy for honey bee health. We hypothesized that deformed wing virus (DWV) and/or sacbrood virus (SBV) would reduce flight performance and that coinfections would have compounding, negative impacts. We identified virus-specific effects; bees with DWV flew shorter distances at slower speeds, whereas bees with SBV flew greater distances at higher speeds. Bees with high virus loads expressed more *heat shock protein 90*, and SBV-infected bees expressed more *octopamine* β*-2 receptor* (*O*β*-2R*). *O*β*-2R* binds octopamine, a “fight or flight” molecule, stimulating metabolic activity, neuromuscular transmission, and movement. To examine relationships between virus infection, octopamine, and flight, we compared the flight performance of DWV-infected bees with octopamine treatment and demonstrated that octopamine negated DWV-associated flight impairment. These findings have organismal-, colony-, and ecosystem-level implications.

## Teaser

Inapparent virus infections differentially affect honey bee flight and have colony- and ecosystem-level implications.

## INTRODUCTION

Viruses infect all living organisms including bacteria, plants, and animals which may result in a range of symptoms or death. The field of virology has primarily focused on viruses that cause symptomatic infections in mammals or plants due to their associated diseases, mortality, and economic losses (*1*, *2*). Insects are one of the largest groups of animals on the planet. They serve as vectors of many human and plant disease causing viruses and host numerous insect-specific viruses that are relatively understudied (*3*). Insect virology has benefited greatly from studies in the fruit fly model *Drosophila melanogaster* and several mosquito genera (i.e., *Aedes*, *Anopheles*, and *Culex*), but relatively few studies have investigated the impact of viruses on their insect hosts (*4*–*6*).

Honey bees (*Apis mellifera*) are eusocial insects that live in colonies of ~30,000 individuals (*7*). Globally, an estimated 112 million managed colonies play a critical role in the pollination of fruit, nut, vegetable, and oil seed crops (*8*). Therefore, high annual honey bee colony losses in some European countries and North America are concerning (*9*–*13*). In the United States, yearly losses averaging ~39% from 2007 to 2023 challenge the commercial beekeeping industry, may lower crop yield, and increase production costs (*12*, *14*–*17*). Honey bee colony deaths are affected by numerous abiotic and biotic factors including agrochemical exposures, nutritional resource availability, parasites, and pathogens, such as viruses (*16*, *18*–*20*). Most honey bee–infecting viruses are picornaviruses, such as deformed wing virus (DWV) and sacbrood virus (SBV), which were named based on the morphological symptoms (i.e., deformed wings and sac-like fluid filled larvae) that may result from infections during development (*21*–*23*). Viruses are readily transmitted within the colony via contact, trophallaxis, and brood care, including larval feeding and removal (*23*, *24*). Several viruses, including DWV, have been associated with reduced colony populations and deaths, particularly in conjunction with high *Varroa destructor* mite infestation, which serve as an amplifying vector for some viruses (e.g., DWV) and a mechanical vector for others (e.g., SBV) (*18*, *23*, *25*, *26*). Recent colony monitoring efforts revealed that adult honey bees often harbor very high virus loads (e.g., ~10^6^ to 10^13^ DWV and/or SBV copies per bee) (*16*, *27*–*31*). While much of the literature suggests that covert infections have negligible impact on individual bee health (*32*–*34*), the impacts of these sublethal, seemingly asymptomatic, virus infections in honey bees have not been well characterized.

Viruses are obligate, intracellular parasites that use host resources for replication. Therefore, we hypothesized that honey bees with high viral burdens may suffer negative physiologic consequences despite appearing asymptomatic and that flight distance may serve as a proxy for individual honey bee health. Honey bee flight performance has been evaluated using roundabout flight mills (*35*–*39*), and some studies indicated that honey bee pathogens reduce flight performance or foraging duration (*31*, *40*, *41*); none quantitatively evaluated the impact of individual and mixed virus infections on flight distance nor examined the impact of coinfections on honey bee health.

## RESULTS

To examine the impact of inapparent virus infections on individual honey bee health, using flight distance as a proxy, we measured the distance flown by virus-infected bees. Honey bees were either mock or virus infected using different doses to establish a range of infection levels observed in natural sublethal infections. At 72 hours postinfection, the flight performance of 4-day-old honey bees was assessed using a flight mill which recorded flight distance, duration, and speed (fig. S1). We conducted five independent experiments with bees from different colonies. Honey bees in this study (*n* = 240) flew up to 5565 m with active flight durations up to 120 min (data S1).

### Honey bee pathogen detection and quantification

The honey bees in this study were obtained from managed colonies that are naturally exposed to commonly occurring pathogens. To reduce the incidence and abundance of confounding virus infections, we used honey bees that emerged in the laboratory setting (*42*–*44*). Pathogen-specific polymerase chain reaction (PCR) was used to detect DWV and SBV, as well as eight additional potential preexisting virus infections and five nonviral pathogens on pooled samples of mock-infected bees for all five experiments (experiments 1 to 5) (data S1 and S2 and fig. S2) (*45*). These analyses determined that honey bees in this study lacked 10 of the 13 pathogens. Most bees had preexisting DWV infections (189 of 229 bees), bees in four experiments (i.e., 1, 2, 4, and 5) had preexisting SBV infections, and 11 bees in experiment 2 were IAPV infected (data S1 and fig. S2). Therefore quantitative PCR (qPCR) was used to determine DWV and SBV abundance in individual bees from all experiments and IAPV abundance in each bee from experiment 2 (data S1 and fig. S2B). Total virus abundance, including both naturally occurring and experimentally introduced virus, ranged from 0 to 9.9 × 10^9^ virus RNA copies/2 μg of total RNA or approximately 0 to 2.6 × 10^11^ virus copies per individual honey bee and correspond to natural levels of virus in adult honey bees (*16*, *26*–*28*, *31*). Virus abundance and gene expression data were obtained by analyzing RNA isolated from honey bee abdomens, since we determined that it was the most representative of whole bee data (fig. S3). Honey bees experimentally infected with DWV, SBV, or coinfected with DWV and SBV had greater virus levels than mock-infected bees (Mann-Whitney *U* = 559, *P* < 0.001) (data S1 and S4).

### DWV and SBV had differential impacts on flight distance

**Fig. 1.**
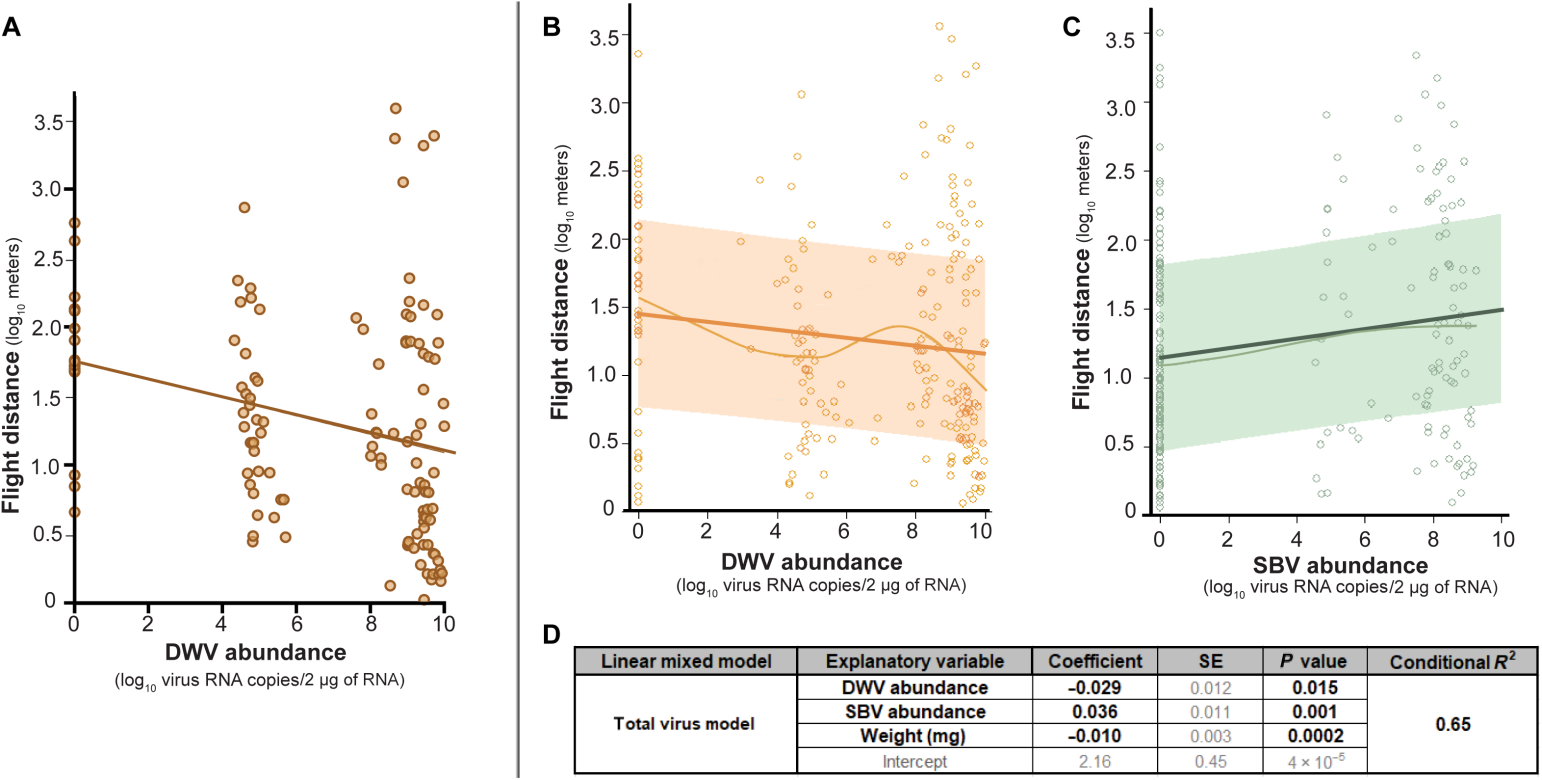
DWV and SBV differentially affect honey bee flight. (**A**) DWV abundance and flight distance in honey bees was negatively correlated (regression coefficient = −0.07, *P* = 0.002); points represent data from bees that either had undetectable virus levels (*n* = 17) or were only infected with DWV (*n* = 111). (**B** to **D**) The relationship between virus abundance and flight distance in honey bees (*n* = 229) infected with DWV and/or SBV was evaluated using a linear mixed model which (B) confirmed the negative relationship between DWV abundance and honey bee flight distance and (C) revealed a positive relationship between SBV abundance and flight distance. Each point represents an individual bee after accounting for random and fixed effects of the best-fit model, the thin line represents the observed trend, the thick line represents the model predictions, and the shaded band represents the 95% confidence interval of the prediction estimates. (D) The best fit linear mixed model included DWV abundances, SBV abundances, and whole bee weight (milligrams) as fixed effects and colony/experiment and flight mill as random effects. The conditional coefficient of determination (*R*^2^) value indicated that the model explained 65% of the variation in honey bee flight distance after accounting for virus abundances, bee weight, and flight mill.

To examine the relationship between honey bee flight distance and virus load, including experimentally introduced and natural virus infections, we first investigated the relationship between DWV abundance and flight distance across all experiments using a simple linear model (*n* = 125 bees without detectable SBV). The simple linear model indicated that DWV has a negative impact on honey bee flight distance (regression coefficient = −0.07, *P* = 0.002) (Fig. 1). Uninfected bees (*n* = 17) flew an average of 58.4 m, whereas bees with low DWV levels (<10^6^ RNA copies/2 μg of RNA; *n* = 35) or high DWV levels (>10^6^ RNA copies/2 μg of RNA, *n* = 72) flew, on average, 21.9 and 14.0 m, respectively (Mann-Whitney, *U* = 836, *P* = 0.002; *U* = 62, *P* = 0.05) (Fig. 1). A simple linear relationship between SBV infection and flight distance could not be determined since just 23 bees were only infected with SBV, and most of them had high infection levels (*n* = 20/23 with >10^6^ RNA copies/2 μg). Experiment 2 had some IAPV-infected honey bees (*n* = 11) that were removed from DWV and SBV analyses, but a comparison of their flight distance compared to mock-infected, IAPV-negative bees (*n* = 21) indicated that they flew shorter distances at lower speeds (fig. S4).

To more accurately describe the relationships between DWV and SBV abundance and flight distance across all experiments, linear mixed models were used to investigate the contribution of other variables. While virus-inoculated honey bees had higher virus loads, the treatment group independent of virus abundance was not a contributing explanatory factor (data S4). Similarly, thorax weight, wingspan, and house identification number (ID) did not improve model quality (data S4 and S5). The inclusion of experiment and flight mill as random effects and whole bee weight as a fixed-response variable improved the model (Fig. 1, fig. S5, and data S4 and S5). The linear mixed model explained 65% of the data variation. Further analysis of the data using the linear mixed model confirmed that DWV abundance had a negative relationship with flight distance and revealed that SBV abundance had a positive relationship with flight distance (Fig. 1). Therefore, their impact was nearly negated in coinfected bees [i.e., the model predicts that bees with DWV and SBV at moderate levels (i.e., 10^5^ copies/2 μg of RNA) would fly 35 m, whereas an uninfected honey bee would fly 32 m]. The model estimates that honey bees with high DWV levels (i.e., 10^9^ DWV RNA copies/2 μg of RNA) would fly 49% shorter distance than bees without DWV and that honey bees with high SBV levels (i.e., 10^9^ SBV RNA copies/2 μg of RNA) would fly 53% farther compared to bees without SBV.

### Virus infections affect flight speeds

**Fig. 2.**
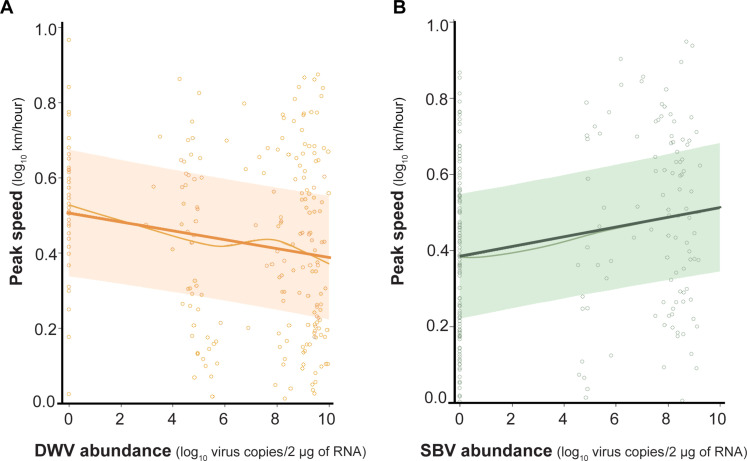
Peak honey bee flight speeds negatively correlated with DWV and positively correlated with SBV. The relationship between peak flight speed, the maximal speed (kilometers per hour) sustained for >10 s, and virus abundance was assessed using linear mixed models. (**A**) Peak flight speed was negatively correlated with DWV abundance (regression coefficient = −0.015, *P* = 0.02) and (**B**) positively correlated with SBV abundance (regression coefficient = 0.19, *P* = 0.001). Each point in the effect plots represents individual bee data, the thin line is the observed relationship, the thick line is the predicted relationship, and the shaded region denotes the 95% confidence interval. The conditional *R*^2^ value indicated that the model explained 42% of the variation in honey bee peak flight speed after accounting for virus abundances, mill, and experiment.

To further evaluate the virus-associated differences in honey bee flight performance, we calculated average flight speed and peak flight speed (i.e., the maximum speed sustained for ≥10 s) and evaluated differences using a mixed effect model (i.e., fixed effect virus abundance, random effect mill, and experiment). DWV levels did not appreciably affect average flight speeds (regression coefficient estimate = −0.004, *P* = 0.54), whereas bees harboring greater SBV levels flew at faster average speeds than bees harboring less SBV (regression coefficient estimate = 0.02, *P* < 0.001) (data S1). In addition to average speed, we compared the maximal peak flight speeds. Peak flight speeds were greater in honey bees with high SBV levels (regression coefficient estimate = −0.15, *P* = 0.02), whereas bees with high DWV levels had lower peak flight speeds (regression coefficient estimate = 0.02, *P* = 0.001) (Fig. 2). There were no detectable differences in active flight durations in DWV, SBV, or total virus levels (regression coefficient estimates, *P* > 0.09). Therefore, differences in flight distance were likely a consequence of flight speed and not attributable to flight time (data S1).

### Honey bees with high DWV levels stopped less frequently to groom

For each flight assay, honey bees were manually instigated to fly up to three times to measure flight to exhaustion rather than motivation for flight. During flight assays, we observed that mock-infected bees stopped for short durations (i.e., <20 s) more often than virus-injected bees. During these breaks in flight, bees attempted to groom themselves free from the flight mill using their mesothoracic legs (fig. S1). This behavior was not observed when the bees were in their houses with the washers affixed to their thoraces.

To quantitatively examine the relative number of short stops bees made during the flight assays, the total number of stops (>1.5 s) was calculated for each flight, and models included the potential impacts of experiment and flight mill. The relationship between virus abundance and flight stop count was assessed using Pearson’s correlation which determined that DWV abundance was negatively associated with the number of stops bees made during the flight assay (regression coefficient estimate = −0.04, *P* < 0.001), whereas SBV abundance was not (regression coefficient estimate = 0.0002, *P* = 0.99, respectively). These results suggest that bees with high DWV levels exerted less energy trying to groom themselves away from the flight mill arm (fig. S6).

### Virus infections result in greater heat shock protein expression

**Fig. 3.**
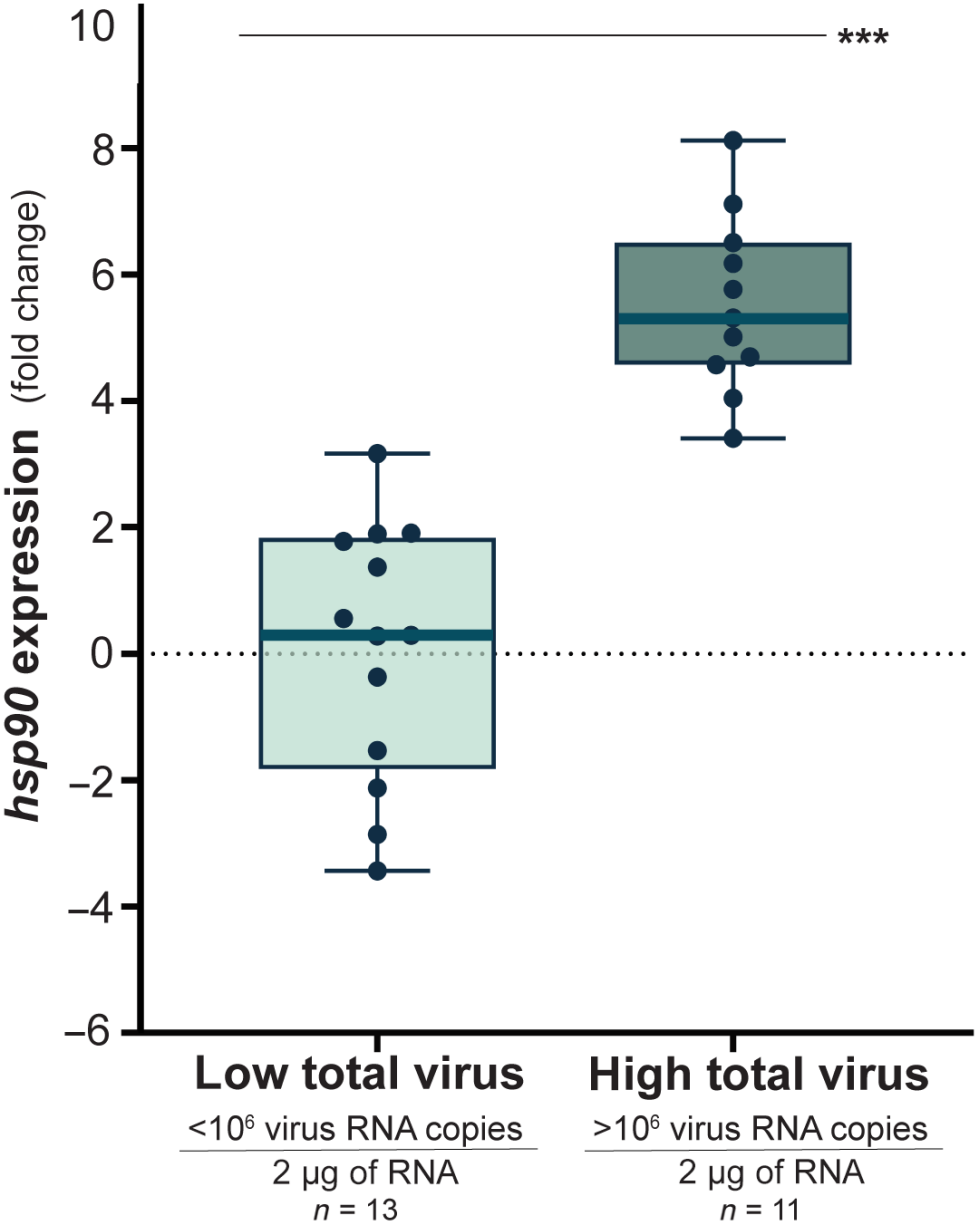
Heat shock protein gene expression and virus abundance are positively associated. *hsp90* expression was greater in bees with higher virus levels (Mann-Whitney, Bonferroni correction, *U* = 0, *P* < 0.001 experiment 2; data from three experiments in fig. S8). Transcript abundance of *hsp90* was normalized to *rpl8*, and fold change was calculated relative to the average expression in mock-infected bees, which had only low levels of preexisting virus (ΔΔCt method). ***P < 0.001.

To further examine the impact of DWV and SBV infection on honey bees, we assessed the relative expression of select genes associated with immune responses and bee health. Specifically, we measured the expression of a gene broadly associated with bee health (*vitellogenin*), genes involved in RNA interference [*dicer* and *argonaute-2* (*ago2*)], and genes involved in the stress-induced heat shock response [*heat shock protein 90* (*hsp90*), *protein lethal*(*2*) *essential for life-like* (*pl2*), and *bee antiviral protein-1* (*bap1*)] in a subset of honey bees to identify trends (data S1) (*23*, *46*). Expressions of *vitellogenin*, *dicer*, *ago2*, *bap1*, and *pl2* were not consistently associated with virus abundance (data S1 and fig. S7), which may be a consequence of mixed DWV and SBV infections, since viruses differentially affect immune gene expression (*47*). In bees coinfected with DWV and SBV, the total virus abundance had a positive relationship with *hsp90* expression in the three experiments examined (Pearson’s correlation, Bonferroni correction, regression coefficient = 0.32, *P* < 0.001) (Fig. 3 and fig. S8).

### SBV increases octopamine receptor expression and octopamine improves flight

**Fig. 4.**
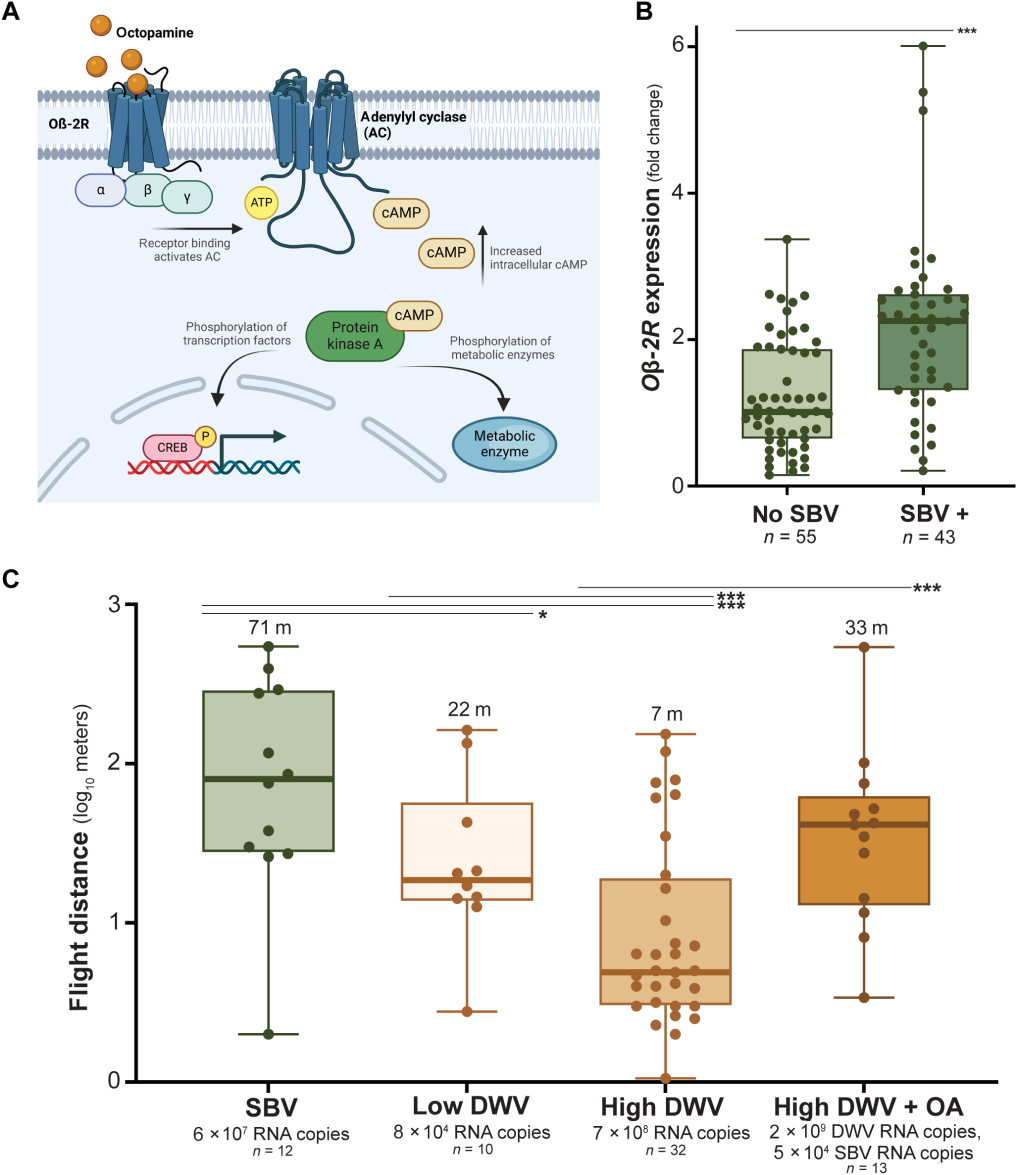
The OA response is associated with greater flight capability. (**A**) Schematic of OA signaling via the Oβ-2R, a G-coupled protein receptor involved in increasing metabolic activity. OA binding of Oβ-2R causes a conformational change that releases the G protein which binds and activates adenylyl cyclase (AC). Activated AC converts adenosine 5′-triphosphate (ATP) to cyclic adenosine 3′,5′-monophosphates (cAMP). Increased intracellular cAMP activates cAMP-dependent protein kinase A (PKA). Activated PKA phosphorylates enzymes and transcription factors associated with elevated metabolism activity including CREB (cAMP response element binding protein). Created in BioRender. Kaku, N. (2025) https://BioRender.com/y8whcnu. (**B**) The expression of *O*β*-2R* was ~1.8× greater in SBV-infected bees compared to those without SBV (Mann-Whitney, Bonferroni correction, *U* = 4.5, *P* < 0.001). (**C**) The flight distance of SBV-infected bees (SBV), mock-infected bees with relatively low DWV-levels (low DWV), and DWV-infected bees with high DWV levels (high DWV) without or with OA co-injection (DWV + OA) was compared; DWV + OA bees flew 79% further distances than bees with high DWV levels, indicating that OA treatment mitigated DWV-associated flight impairment (virus abundance is shown as the average RNA copy number per 2 μg of RNA; individual bee data in data S1). ***P < 0.001. P, phosphate group.

Octopamine (OA), a “fight or flight” stress molecule in invertebrates, binds and activates OA receptors, including the OA β-2 receptor (Oβ-2R). Increased Oβ-2R activity results in increased metabolic activity and enhanced neuromuscular signaling (Fig. 4A) (*48*–*51*). We assessed *O*β*-2R* expression in virus infected bees and determined that expression was positively correlated with SBV infection levels (*P* < 0.05) but not with DWV abundance in bees with only DWV (experiment 3, *n* = 21; Pearson’s correlation, *P* = 0.81) nor in coinfected bees. The relationship between *O*β*-2R* expression and SBV was bimodal. Therefore, we compared *O*β*-2R* expression in bees with or without SBV infections and determined that SBV-infected bees expressed more *O*β*-2R* than SBV-negative bees (Mann-Whitney, Bonferroni correction, *P* < 0.05) (Fig. 4B and fig. S9).

To confirm the potentially flight-enhancing impact of the OA response in honey bees, we injected a subset of bees in experiment 5 with moderate levels of the flight-impairing virus, DWV (i.e., 3.5 × 10^4^ RNA copies) with or without a biologically relevant dose of OA (2 μl of 10 mM). Despite harboring higher levels of DWV, OA-treated bees (*n* = 13) flew greater distances than untreated DWV-infected bees (*n* = 32) (i.e., geometric mean distance of 33 m versus 7 m, respectively) (Fig. 4C). We used the linear mixed model to predict the flight distance for bees with 6 × 10^7^ SBV RNA copies per 2 μg RNA, and the expected distance was 70 m, which is very similar to the observed distance of 71 m (Fig. 4C). Since the OA-treated bees also had low levels of SBV due to a preexisting infection, we used the linear mixed model to predict the flight distance for bees with the virus levels in that treatment group. The model predicted that DWV + OA bees would fly 32% further distance than high DWV bees due to preexisting SBV. We observed that DWV + OA bees flew 79% further distances than bees with high DWV levels. Together, these data indicate that OA may mitigate DWV-associated flight impairment and point to a physiological explanation for the differential impact of SBV and DWV on honey bee flight performance. Future studies that evaluate the impact of OA on flight performance in bees without virus infection are necessary.

## DISCUSSION

The impact of virus infections on the flight performance of birds, bats, and insects is underexplored but may have important ecological implications (*52*–*54*). Here, we describe two honey bee–infecting iflaviruses, DWV and SBV, that have differential impacts on honey bee flight performance. DWV and SBV infections of adult bees are prevalent and abundant, DWV has been associated with poor colony health and longevity, and both DWV and SBV are associated with developing bee deaths (*16*, *26*–*28*, *30*, *34*). Impairment or death of too many individual honey bees leads to colony death, since thousands of individuals are required to maintain the superorganism (*7*). Recent high annual losses of honey bee colonies invigorated research on the abiotic and biotic factors affecting colony health, including viruses—which have been associated with colony deaths. Virus monitoring efforts revealed that most honey bees in North America and Europe are infected with DWV by mid-summer, and many harbor high virus levels (e.g., ~10^6^ to 10^13^ DWV and/or SBV copies per bee) (*16*, *27*–*31*), but the potential impacts of these infections on honey bee health was unknown. We used flight performance as an indicator of overall bee health. Previous studies assessed the flight performance of bees of multiple ages in flight chambers and determined that, while forager bees (~ 2 to 3 weeks posteclosion) exhibited better flight performance, nurse bees fly as young as 3 days posteclosion (*55*, *56*). The absence of typically aged foragers causes younger bees to fly precociously (*56*–*59*). To our knowledge, our study is the first to examine flight performance of individual, age-matched honey bees that were experimentally infected with DWV and/or SBV and tested for potential confounding infections. Previous studies used optical entrance counters or radio frequency identification–tagged honey bees and found that honey bees that were wounded or infected with the microsporidia *Nosema ceranae* and/or DWV took fewer trips, and DWV infection was associated with precocious foraging (*40*, *41*). While these results were intriguing, neither flight performance (i.e., distance or speed) nor virus abundance was assessed, and potential confounding infections were not considered (*40*). More recently, data from video monitored colonies indicated that IAPV inoculation reduced honey bee social behaviors but was not associated with flight trip frequency nor duration (*60*). The single prior study that used flight mills to evaluate flight performance of infected honey bees determined that DWV and/or *N. ceranae*–infected bees flew shorter distances than uninfected bees (*31*). However, only 8.6% of the variation in flight distance was associated with infection, likely because there were relatively few bees with DWV that lacked *Nosema* and even fewer that were negative for both pathogens, the bees varied in age, and the impacts of potentially confounding, preexisting infections were not assessed (*31*).

Co-occurrence of virus infections that do not cause overt morphological symptoms in honey bees is common (*16*, *30*, *45*, *61*, *62*). In this study, 78% of the mock-infected honey bees had detectable preexisting virus but lacked morphological symptoms (i.e., 62 of 79 bees). We hypothesized that virus infections would negatively affect honey bee flight capability and that viral coinfections would have a compounding, negative influence on flight. We infected age-matched honey bees with DWV and/or SBV, using doses similar to other caged bee studies and in line with field data (*16*, *27*–*31*, *44*). We determined that DWV and SBV had differential impacts on honey bee flight performance, which may partially explain previous inconsistent results and the higher number of studies that correlate DWV with colony losses (*18*, *23*, *25*). We report that honey bees with high DWV loads flew 49% shorter distances at lower speeds than uninfected bees and exhibited a lower frequency of energetically taxing grooming behaviors. Unexpectedly, we observed a positive relationship between flight capability and SBV levels. During coinfections of DWV and SBV, SBV masks the negative consequences of DWV infection on honey bee flight. This result alone does not support an overall beneficial role of SBV infections, which are often pathogenic (*63*, *64*).

We examined the expression of genes that serve as biomarkers of bee health, including genes involved in antiviral defense and stress responses. Expression of these genes did not appreciably differ between DWV and SBV infections, but since pupae exhibit differential responses (*47*), we expect that future full-transcriptome analyses of infected adult bees will reveal genes and pathways that correlate with differences in flight performance. In previous studies, we determined that virus-infected honey bees had higher expression of genes involved in metabolism and the heat shock stress response (HSR) pathway compared to mock-infected bees and that the HSR is involved in reducing model virus infection levels in honey bees (*43*, *46*). While the role of the HSR in honey bees infected with naturally occurring viruses requires further investigation, we found that the expression of *hsp90* correlated with total virus abundance. Honey bee body temperature is elevated to levels that may activate a heat shock response (*31*, *65*–*67*), and one study determined that heat stressed colonies had lower DWV levels (*68*). Therefore, we will evaluate the relationship between HSR, flight, and antiviral defense in future studies.

In this study, we determined that expression of *O*β*-2R* was greater in SBV-infected bees, which also exhibited enhanced flight performance. OA is broadly referred to as the fight or flight molecule in insects due to its behavioral influence in the context of external stressors (*69*, *70*). Activation of this pathway results in increased metabolic activity and enhanced neuromuscular signaling (*48*, *49*). Experimental introduction of OA in young bees also induces lipid mobilization and initiation of the nurse bee to forager transition (*71*, *72*). Previous work also determined that OA injection caused honey bees in small chambers to fly for greater periods of time in flight, although specific flight metrics were not measured (*72*). Both hemolymph OA levels and *O*β*-2R* expression increase as bees become more adept to flight (*48*). Our results suggest that SBV infection induces the Oβ-2R–regulated pathway, which may account for the increased flight distance and speeds of SBV-infected honey bees and that OA mitigates DWV-associated flight impairment.

Flight is the most metabolically costly honey bee behavior (*36*, *38*, *73*). In natural settings, these energetically taxing infections coupled with reduced foraging capabilities may result in insufficient resources to sustain the colony, particularly if virus-infected bees require more food (fig. S10 and data S7) (*23*, *74*, *75*). Honey bees fly to provision the colony with nectar and pollen resources. While foraging, bees pollinate plants in both wild and agricultural landscapes including crops valued at $175 billion per year globally (*76*, *77*). Investigations of alarmingly high annual colony deaths indicate that multiple factors, including viruses such as DWV, contribute to these losses (*18*, *23*, *25*, *78*). Therefore, we estimated the colony-level impact of asymptomatic DWV infections using an equation that accounted for the reduced flight distance of highly infected foragers and an estimated percentage of virus-infected foragers, which was based on empirical data and statistical models (data S6) (*79*). Our analysis indicates that DWV infections may hinder the foraging capacity of the colony, resulting in less food for the colony and increasing the chances of colony death due to nutritional stress.

In addition to the impact of virus infection on individual bees and colony health, the data presented herein may also have ecosystem level impacts. Honey bees with reduced flight capabilities may pollinate fewer plants within a limited range. Bee-infecting viruses have a broad host range and can be shared between sympatric species via floral resources, although the extent of virus overlap varies and transmission events may be rare (*80*–*83*). Healthy honey bees may forage up to 5 km from their colonies but often focus on more proximate resources, whereas other bee species forage closer to their nest (e.g., bumble bees, ~500-m range). Our results indicate that the likelihood of virus transmission between highly DWV-infected honey bees and coforaging bees would be greater near the hive, while the overall zone of virus transmission may be reduced. Conversely, the zone of SBV transmission may be larger. Our results may also partially explain higher prevalence of DWV in native/wild bee species obtained from some sites located near managed honey bee colonies (*83*–*85*). Collectively, the data presented herein demonstrate that the impacts of inapparent honey bee virus infections are virus specific and indicate how viral infections affecting individual behavior and health may have colony- and ecosystem-level impacts.

## MATERIALS AND METHODS

Additional details are included in the Supplementary Materials.

### Honey bees

Honey bees (*A. mellifera*, primarily *carnica*) were obtained from colonies maintained using standard apicultural practices on Montana State University’s Horticulture Farm. Independent experiments were performed with honey bees from different colonies to obtain robust results from genetically diverse honey bees (data S1). Honey bees for experiments were obtained from frames 1 to 2 days before experiments, cohoused in modified deli containers (*n* = 5 to 11 per house), maintained at 32°C in an incubator, and fed sucrose patties and water ad libitum (*43*). The sucrose consumed was measured (i.e., 8 to 11 bees per house) for three of the five experiments (experiments 3, 4, and 5) (data S7 and fig. S10). Honey bees in a parallel study were fed 50% sucrose syrup, and sucrose consumption over the 72-hour study was measured (data S7 and fig. S10). Together, these data indicate that differences in sucrose consumption did not scale with differences in flight performance.

### Virus stock preparation

DWV, SBV, and coinoculums (DWV and SBV) were propagated in white-eyed honey bee pupae that were injected with 4.0 × 10^6^ DWV RNA copies, 3.5 × 10^4^ SBV RNA, or 3.5 × 10^4^ DWV and 3.0 × 10^4^ SBV copies in 2 μl between the second and third abdominal tergites using a Harbo syringe and a disposable borosilicate needle made from modified capillary tubes. Pupae were maintained in a humidified 24-well plate at 32°C for 7 days postinjection and then homogenized in a 2-ml microfuge tube with a sterile metal 3-mm bead in 1 ml of 1× phosphate-buffered saline (pH 7.4) using the Tissue Lyser II (QIAGEN) at 30 Hz for 2 min. Homogenates were centrifuged at 14,000*g* for 15 min at 4°C, and the supernatant was transferred to a fresh tube. The virus-containing lysates were filtered through a 0.45-μm filter and then a 0.22-μm filter to remove large particles and nonviral microbial contaminants. RNA was isolated from the virus-containing filtrate (20 μl) using TRIzol reagent (Thermo Fisher Scientific) according to the manufacturer’s instructions; RNA was quantified using a NanoDrop 2000c Spectrophotometer, cDNA was synthesized with reverse transcriptase, and virus RNA copies, including both genomes and transcripts, were quantified from cDNA using qPCR. The inoculum was tested for copurifying/contaminating viruses via PCR (data S2 and fig S2). RNA copy concentration in the purified virus stock was quantified relative to a standard curve using qPCR (data S3). DWV genome consensus sequences were assembled from short-read sequencing data obtained from sequencing RNA isolated from the inoculum (DWV-lab 2024; GenBank, PV821422), a naturally infected honey bee (DWV-natural MT2024; GenBank, PV821421), and an experimentally infected honey bee (DWV-exp infect MT2024; GenBank, PV821420); these sequences shared ≥90% nucleotide identity (Supplementary Materials).

### Experimentally introduced honey bee virus infections

Honey bees were infected with DWV using previously described methods (*43*). Briefly, age-matched bees were cold anesthetized at 4°C for 10 min and infected via intrathoracic injection using a Harbo syringe with 3.5 × 10^3^, × 10^4^, and × 10^5^ DWV RNA copies, 3.5 × 10^4^ SBV RNA copies, or 3.5 × 10^4^ DWV RNA copies + 9.1 × 10^3^ SBV RNA copies in 2 μl of 10 mM tris HCl buffer (pH 7.5). Mock-infected bees were injected with 2-μl buffer. DWV inoculum was diluted into OA (10 mM) for experiment 5. The DWV doses used in this study were similar to other studies and resulted in a range of virus levels that are commonly found in naturally infected bees (*16*, *27*–*31*). Immediately following injection, bees involved in flight assays had #000 steel washers affixed to their thoraces using rubber cement; washers had an outside diameter of 0.19 cm and weighed 4.3 mg, less than half of an average pollen load (*7*).

### Flight mill

The general design of the flight mills used in this study (fig. S1) was based on flight mills used in previous honey bee studies (*35*, *36*, *38*). Unique features of the flight mills described herein include a flight arm that allows vertical movement, in addition to horizontal flight, which may enable more natural flight patterns and a magnet to tether and remove bees from flight arms with minimal damage.

### Honey bee flight assay

Honey bees (3 days postinjection and 4 days posteclosion) were immobilized via incubation at 4°C for ~12 min and tethered to flight mills; counterweights were adjusted for each bee. Temperature and humidity were maintained (23°C and 31 ± 4% relative humidity). Flight was instigated by tapping the flight arm downward. Flight was reinitiated up to three times before bees were considered “exhausted,” and the data collected were representative of “total flight” capability (data S8). Immediately after flight, bees were stored at −80°C. Flight distance calculations were based on detector counts. The encoder disc within the central rod base of each flight mill has eight light-obstructing bars and eight empty slots; therefore, the detector counts 16 light changes per full revolution. The flight arm is 27.94 cm long; therefore, a full revolution is 88 cm [i.e., circumference calculated using C = 2π(*r*)]. The redlight sensor was attached to a modified single-board computer (i.e., Raspberry Pi) containing a microSD card to store data (data S8). Active flight durations were calculated via redlight signal interruptions every 0.5 s, which can be used to determine the time each bee was actively flying. Active flight duration was automatically calculated as the sum time of any 5.5-cm movement (one encoder count) (data S9). Flight stop count was calculated using batch file script and defined as any stop in movement that exceeded 1.5 s, with three stop counts subtracted to account for manual flight instigations (data S9).

### Honey bee measurements

Individual frozen honey bees were weighed three times and averaged to ensure accuracy (data S5). Bees were dissected into head, thorax, and abdomens, weighed separately, and stored at −80°C. Bee weight negatively correlated with flight distance (Fig. 1 and fig. S5), likely a consequence of less efficient weight distribution (*86*), as wingspan did not scale with whole bee weight nor affect flight distance (*n* = 26, regression coefficient estimate = 0.1, *P* = 0.3) (data S8). Honey bee forewings were extended, pinned, and measured from the tip of the left forewing to the tip of right forewing. Since wingspan did not correlate with bee weight nor flight distance, it was not included in linear mixed models (data S5).

### Honey bee RNA isolation

RNA was isolated from the abdomen of all honey bees. To ensure that data from abdomen samples were representative of the whole bee, we extracted RNA from the head, thorax, and abdomen of select honey bee samples and determined that both the RNA yield (i.e., nanogram RNA per milligram tissue) and virus abundance was consistent across the different individual bee body segments and representative of the entire bee (fig. S4 and data S10). Individual segment samples were homogenized in sterile water (abdomens/thoraces, 400 μl; heads, 200 μl) with one sterile steel bead (4.5 mm) using a QIAGEN TissueLyzer for 2 min at 30 Hz. Lysates were centrifuged at 4°C for 5 min at 7500*g*. RNA was isolated from the supernatants using equal volumes of TRIzol reagent according to the manufacturer’s instructions. RNA concentration and quality were assessed on a NanoDrop 2000 Spectrophotometer (Thermo Fisher Scientific), and RNA samples were stored at −80°C. Virus quantification from abdomen samples was reflective of whole bee data (fig. S4 and data S10).

### Reverse transcription/cDNA synthesis

Reverse transcription reactions were performed by incubating 2 μg of total RNA, 200 U of Moloney murine leukemia virus reverse transcriptase (M-MLV) reverse transcriptase, and 500 ng of random hexamer primers in 25-μl reactions for 1 hour at 37°C, according to the manufacturer’s instructions. cDNA was diluted with sterile water (1:2), and 2 μl was used for PCR or qPCR analysis.

### Polymerase chain reaction

PCR was performed according to the standard methods to test for preexisting virus infections since bees were obtained from colonies which are subject to naturally occurring infections (*27*, *43*). Briefly, a 2-μl cDNA template was combined with 10 pmol of each forward and reverse primers (data S2) and amplified with ChoiceTaq polymerase according to the manufacturer’s instructions using the following conditions: 95°C for 5 min; 95°C for 30 s, 57°C for 30 s, 72°C for 30 s, 35 cycles, followed by final elongation at 72°C for 4 min. PCR products were assessed by gel electrophoresis (1.5% agarose with SYBR safe) and visualized using a Syngene U:Genius 3 imaging system (fig. S2). All products were previously verified by sequencing (*27*). Analysis of pooled cDNA samples from mock-infected honey bees from each of these experiments revealed that many bees had preexisting DWV and SBV infections; therefore, qPCR was used to assess their abundance in all individual samples (data S2). The qPCR primers targeting DWV bind to both DWV-A (GenBank, AY292384) and DWV-B (GenBank, MN565036), as well as numerous other DWV sequences on National Center for Biotechnology Information including the consensus sequences of the DWV inoculum and naturally occurring DWV sequenced as part of this study (Supplementary Materials). Experiment 2 also included IAPV-infected bees, and levels were quantified (data S2 and fig. S2) (*45*).

### Quantitative PCR

qPCR was used to analyze virus abundance and relative abundance of honey bee transcripts. All qPCR reactions were performed in triplicate with 2 μl of cDNA template. Each 20-μl reaction contained 1× ChoiceTaq Mastermix, 0.4 μM each forward and reverse primer, 1× SYBR Green (Life Technologies), and 3 mM MgCl_2_. A CFX Connect Real Time instrument (Bio-Rad) was used for the following thermoprofile: preincubation 95°C for 1 min followed by 40 cycles of 95°C for 10 s, 58°C for 20 s, and 72°C for 15 s, with a final melt curve analysis at 65°C for 5 s to 95°C. To quantify viral copy numbers in the samples, plasmid standards [i.e., virus-specific qPCR amplicons cloned into the pGEM-T (Promega) vector and sequence verified] for each virus were used as templates, ranging from 10^3^ to 10^9^ copies per reaction for each standard curve (data S3). The honey bee gene *rpl8* was amplified in triplicate for each sample for comparison and to assess cDNA quality (data S2). Reactions without template were used as negative controls. qPCR specificity was verified by melt point analysis, gel electrophoresis, and sequencing (*27*). For each honey bee sample, the starting quantity (SQ) for each well with a cDNA template representing 80 ng of total honey bee RNA was calculated on the basis of the standard curve and subtracting the average SQ of the no template control reaction (i.e., <600 copies). Values below the limit of detection for qPCR (i.e., <1000 RNA copies/2 μg of RNA) were listed as 0. Virus abundance was reported as virus RNA copies, including genomes and transcripts, per 2 μg of total RNA (i.e., per reverse transcription reaction) and ranged up to 9.9 × 10^9^ virus RNA copies/2 μg of RNA. Comparison of virus abundance levels using estimated copy numbers per 2 μg of RNA allowed inclusion of values of 0; using the ΔΔCt method to calculate virus levels in bees with no detectable virus would inaccurately result in “missing” data and consequently skew model interpretations. Relative host gene expression was calculated using the average ΔΔCt method where ΔCt was determined by subtracting *rpl8* Ct from the gene of interest Ct. Average ΔCt was calculated by the mean ΔCt of the buffer-injected bees, which harbored the lowest virus levels in each experiment. The ΔΔCt was calculated by subtracting the average control ΔCt, and fold change was determined by the equation 2^- ΔΔCt^ (data S1).

### Statistics

Statistical analyses were performed in R 4.1.2. Log transformations and nonparametric tests were performed as necessary.

### Linear relationship between virus abundance and flight distances

The relationship between flight distance, virus abundance, and other factors was assessed using linear mixed models from the lmer4 package (*87*). Assumptions for linear model analyses were verified by diagnostic plots and histograms. Various models evaluating individual honey bee weight, individual honey bee ID, colony ID, honey bee house ID, day of individual bee frame emergence, treatment group, and flight mill ID as random effects were considered with maximum likelihood when random effects varied or restricted maximum likelihood when fixed effects varied, in addition to Akaike information criterion corrected and Bayesian information criterion values (data S4). The best model with flight distance as the response variable included virus abundance (log_10_ virus copies/2 μg of RNA) as a fixed response and mill ID and colony/experiment ID as random effects. The best-fit linear mixed model equations for DWV and total virus abundance can all be described as follows: [*y*_ij_ = β_0_ + β_1_*X*_1ij_ + β_2_*X*_2ij_ + *z*_1_*u*_i_ + *z*_2_*u*_j_ + ε_ij_], where *y*_ij_ = log_10_ (1 + distance), *X*_1ij_ = log_10_ (1 + DWV abundance), *X*_2ij_ = log_10_ (1 + SBV abundance), *z*_1_*u*_i_ = mill ID, and *z*_2_*u*_j_ = colony/experiment. Model assumptions were assessed using the performance package. To predict values using the linear mixed model, input values were compared via the predict function in the stats package in R using average whole bee weight of 84.3 mg.

### Figures

All figures were generated through Prism 9 software for windows using version 9.4.1, in RStudio using version R 4.1.2, in BioRender, and Adobe Illustrator.

## Supplementary Materials

### The PDF file includes:

Supplementary Text Figs. S1 to S10 Legends for movies S1 to S3 Legends for data S1 to S10 References
