# Supplementary material for: Inapparent virus infections differentially affect honey bee flight
Source: Sci Adv. 2025 Aug 8;11(32):eadw8382. doi: 10.1126/sciadv.adw8382 (PMC12333691; doi:10.1126/sciadv.adw8382)
Supplement: Supplementary file 1 — Supplementary Text Figs. S1 to S10 Legends for movies S1 to S3 Legends for data S1 to S10 References [file sciadv.adw8382_sm.pdf]

Science Advances
AAAS

Supplementary Materials for

Inapparent virus infections differentially affect honey bee flight

Naomi G. Kaku *et al.*

Corresponding author: Michelle L. Flenniken, michelle.flenniken@montana.edu

Sci. Adv. **11**, eadw8382 (2025)
DOI: 10.1126/sciadv.adw8382

The PDF file includes:

Supplementary Text
Figs. S1 to S10
Legends for movies S1 to S3
Legends for data S1 to S10
References

Other Supplementary Material for this manuscript includes the following:

Movies S1 to S3
Data S1 to S10

Supplementary Text

Honey bees

Honey bees (*Apis mellifera,* primarily *carnica*)*,* were obtained from colonies maintained using standard apicultural practices on Montana State University's Horticulture Farm. Female adult bees were obtained from brood frames that were collected 1-2 days prior to each experiment and maintained at 32˚C in a laboratory incubator. Bees were housed in modified deli-containers (n=5-11 bees per container) at 32˚C and fed sucrose patties and water *ad libitum* (*43*). In a parallel study, honey bees were fed 50% sucrose syrup and sucrose consumption over the 72-hour study was measured (data S6). Independent experiments were performed with honey bees from different colonies to obtain robust results from genetically diverse honey bees.

Virus stock preparation

Deformed wing virus (DWV) (GenBank PV821422), sacbrood virus (SBV) (PV788228), and co-inoculums (DWV+SBV) were propagated in white-eyed honey bee pupae collected from a frame of capped brood and incubated at 32°C for 24 hours (*44*). Pupae were injected with $4.0x10^6$ DWV RNA copies, $3.5x10^4$ SBV RNA copies, or $3.5x10^4$ DWV and $3.0x10^4$ SBV copies in 2 µL between the 2nd-3rd abdominal tergites using a Harbo syringe and a disposable borosilicate needle made from modified capillary tubes. Post-injection, pupae were maintained in a humidified 24-well plate at 32°C for 7 days post-injection. Pupae were homogenized in a 2 mL microfuge tube with a sterile metal 3 mm bead in 1mL 1x PBS pH 7.4 using the Tissue Lyser II (Qiagen) at 30 Hz for 2 min. Homogenates were centrifuged at 14,000xg for 15min at 4°C and supernatant was transferred to a fresh tube. The virus-containing lysates were filtered through a 0.45 µm filter, then a 0.22 µm filter to remove large particles and non-viral microbial contaminants. RNA was isolated from the virus-containing filtrate (20 µL) using TRizol reagent (Thermo) according to manufacturer's instructions; RNA was quantified using a NanoDrop 2000c Spectrophotometer, cDNA synthesized with reverse transcriptase, and virus RNA copies, including both genomes and transcripts, were quantified from cDNA using qPCR. The inoculum was tested for copurifying / contaminating viruses (AnBV-1, AmFV, ABPV, BQCV, CBPV, IAPV, KBV, LSVs, and SBV) via PCR (data S2). Only DWV and SBV were detected in the virus inoculums (i.e., only DWV detected in DWV inoculums, SBV in SBV inoculums, and DWV+SBV in the mixed inoculum) (fig. S2). RNA copy concentration in the purified virus stock was quantified relative to a standard curve using qPCR (data S3).

Deformed wing virus stock sequencing

The DWV virus stock utilized in this study DWV-lab 2024 (GenBank PV821422) shares 94% nucleotide identity with DWV-A PA (AY292384) (data not shown). To obtain DWV-lab 2024 consensus sequence, a virus augmented sequencing library was prepared from the DWV virus stock using previously described methods (*82*). In brief, to enhance the amount of virion protected/encapsidated full length DWV genomic RNA, a dilution of the DWV virus stock (150 µL) was treated with 50 U Benzoase (Sigma) and 40 U RNase I (Thermo) in total reaction volume of 250 µL, incubated at 37°C for 1.5 h, then extracted with Trizol reagent according to the manufacturer's instructions. Glycogen (Thermo) was added at a concentration of 20 µg to aid in nucleic acid precipitation. RNA quality and abundance were estimated by NanoDrop and Qubit. RNA was sent to the Roy J. Carver Biotechnology Center at the University of Illinois for library preparation and paired-end sequencing on an Illumina NovaSeq sequencer. An Illumina paired-end sequencing library (2x150 bp) was prepared without polyA selection or

rRNA removal steps since presumably the majority of rRNAs and the cytoplasmic mRNAs would have been degraded by RNAse treatments. The reads were processed as previously described (*82*).

In brief, FastQC was used to view qualities both before and after further quality trimming with Trimmomatic-0.39 (*88*). Trimmed paired-end reads were assembled using Trinity 2.15.2 (*89, 90*), consensus sequences were generated using Geneious Prime version 2024.0, www.geneious.com), and nucleotide alignments were performed using NCBI's blastn (*91*). A consensus genome sequence of the DWV inoculum (DWV-lab 2024, GenBank PV821422) was generated from ~ 3,000,000 reads, >145 nt long with a mean coverage of 62,000x. DWV-lab 2024 shares 94.7% nucleotide identity with DWV-A (GenBank AY292384) and 89.8% nt identity with DWV-B (GenBank OR497373 DWV-B 13E-21). A DWV virus consensus sequence was also assembled from a poly-A selected transcriptome sequencing library from a mock-infected honey bee that had naturally acquired DWV infection (DWV-natural MT2024, GenBank PV821421) and an experimentally DWV-infected honey bee (DWV-exp infect MT2024, GenBank PV821421). These sequences were assembled from short-read data (i.e., ~25 million reads >145 bp with a mean coverage 479,580x and ~16 million reads >145 bp and ~263,699x, respectively) and represent the RNA quasispecies circulating in the infected bees. Importantly, the qPCR primers used in this study bind both of these DWV consensus sequences, which are ~ 92% identical to each other at the nucleotide level.

<u>Experimentally-introduced virus infection of honey bees</u>
Honey bees were infected with DWV virus using previously described methods (*43*). In brief, age-matched bees (~24 h post-emergence) were cold anesthetized at 4°C for 10 min and infected via intra-thoracic injection using a Harbo syringe and microcapillary borosilicate glass needles with 3.5×10^3 , $\times 10^4$, $\times 10^5$ DWV RNA copies, 3.5×10^4 SBV RNA copies, or 3.5×10^4 DWV RNA copies and 9.1×10^3 SBV RNA copies in 2 µL10 mM Tris HCl buffer, pH 7.5. Mock-infected bees were injected with 2 µL buffer. Octopamine injections were suspended at 10 mM in DWV inoculum. The DWV dose utilized in this study was similar to other studies and resulted in a range of virus-levels that are commonly found in naturally infected bees (*16, 27-30, 31*). Immediately following injection, bees involved in flight assays had #000 steel washers affixed to their thoraces; washers had outside diameter of 0.19 cm and weighed 4.3 mg, less than half of an average pollen load (*7*). After injection/manipulation, bees recovered at room temperature within five minutes and were maintained at 32°C until flight assays were performed.

<u>Flight mill</u>
The general design of the flight mills used in this study (fig. S1) was based on flight mills used to evaluate the impact of field acquired pathogens (i.e., DWV and *Nosema*) or experimentally provided insecticide in honey bees (*35, 36, 38*). Unique features of the flight mills described herein include a flight arm that allows vertical movement, in addition to horizontal flight, which may enable more natural flight patterns and a magnet to facilitate may tether and remove bees from flight arms with minimal damage. Attached to the top base arm and nested into the red-light sensor is a 3D-printed encoder wheel which contains 8 light-obstructing bars and 8 empty slots. As the top base rod rotates, the encoder wheel also rotates and interrupts the redlight, which is detected by a redlight sensor as a 'count'. The redlight sensor was attached to a modified single-board computer (i.e., Raspberry Pi) containing a microSD card to collect and store data.

Honey bee flight assay and calculations

After 72-hours, adult worker bees that were either mock- or DWV infected were immobilized by incubating each cage (n=5-11 bees/group) for ~12 minutes at 4°C, tethered to flight mills, and counterweights were adjusted to counter individual bee weights and verified by measuring flight arm to axle angle of 90 degrees with a protractor. The flight room temperature was maintained at 23°C with 31±4% relative humidity, and fluorescent overhead lights. Approximately 1-5 minutes after tethering, flight was instigated by tapping the flight arm downward. Vertical movement did not contribute to distance calculations. Flight was re-initiated up to three times before bees were considered 'exhausted' and the data collected was representative of 'total flight' capability. After the flight assay, bees were frozen and stored at -80°C.

The encoder disc within the central rod base of each flight mill has 8 light-obstructing bars and 8 empty slots, therefore the detector counts 16 light changes per full revolution. The flight arm is 27.94 cm long, therefore the distance flown by a bee in each full revolution is 88 cm (i.e., the circumference calculated using $C=2\pi(r)$). Therefore, to calculate the distance flown by each bee, the number of counts detected is divided by 16 to determine total revolutions, which is multiplied by the circumference of one revolution (88 cm) and divided by 100 to calculate the total distance flown in meters.

Each flight mill records the number of redlight signal interruptions detected every 0.5 seconds, which can be used to determine the time each bee was actively flying. Active flight duration was automatically calculated through python script as the sum time of any 5.5 cm movement (one encoder count) (data S9).

To measure energy expenditure not associated with flight distance, honey bee flight stop count was calculated from flight mill data. Flight stop count was automatically calculated using batch file script and defined as any stop in movement that exceeded 1.5 seconds, with 3 stop counts subtracted to account for manual flight instigations. Honey bees with negative flight stop count values were due to bees failing to initiate flight after manual instigation (data S9). To normalize flight stop count, data was $\log_{10}$ transformed and a standard value of 3.001 was added to each to retain any negative values during analyses.

Honey bee measurements

To assess potential relationships between honey bee weight and flight distance, RNA yield, and DWV abundance, individual frozen honey bees were weighed three times and averaged to ensure accuracy (average whole bee weight 90.8 mg); the average weight of a steel washer was subtracted from each whole bee weight (data S5). Then bees were dissected into head, thorax, and abdomens, weighed separately, and stored at -80°C. We weighed the head, thorax, and abdomen segments of a small subset of bees (n=15) and determined that most of the variation in whole bee weight was due to the abdomen weights (data S10). Honey bee weight negatively correlated with flight distance (Fig. 1, fig. S5), which was likely a consequence of less efficient weight distribution (*13*) as wingspan did not scale with whole bee weight and wingspan alone did not appreciably impact flight distance (n=26, regression coefficient estimate=0.1, p=0.3) (data S8).

To measure bee wingspans, forewings were extended, pinned, and measured from tip of the left forewing to the tip of right forewing. For a subset of individual honey bees, comparison of coefficient significance indicated no relationship between wingspan and honey bee weight nor flight distance therefore wingspan was not considered in model selection (data S5).

Honey bee RNA isolation and purification

RNA was isolated from the abdomen of all honey bees. To ensure that data from honey bee abdomen samples were representative of the whole bee (12, 25), we extracted RNA from the head, thorax, and abdomen of select honey bee samples and determined that both the RNA yield (i.e., ng RNA/mg tissue) and virus abundance was consistent across the different individual bee body segments and representative of the entire bee (fig. S4, data S10). Individual segment samples were homogenized in sterile water (abdomens/thoraces 400 μL, heads 200 μL) with one sterile steel bead (4.5 mm) using a Qiagen TissueLyzer for 2 min at 30 Hz. Lysates were clarified by centrifugation (7,500× *g* at 4 °C for 5 min). RNA was isolated from lysates using equal volumes of TRizol reagent according to manufacturer's instructions. RNA concentration and quality was assessed on a Nanodrop 2000 spectrophotometer (Thermo Fisher) and RNA samples were stored at −80°C. Quantification of DWV and SBV abundance via qPCR in individual bee segments (n=15) indicated that estimating virus copy numbers based on head and thorax samples tended to underestimate virus abundance (fig. S4, data S9). Virus quantification was more consistent in abdomen samples and there were no false-negative results (fig. S4 and data S10), therefore, abdomen virus abundance data was used in the linear models.

Reverse transcription / cDNA synthesis

Reverse transcription reactions were performed by incubating 2000 ng total RNA, 200U M-MLV reverse-transcriptase and 500 ng random hexamer primers in 25 μL reactions for 1h at 37°C, according to the manufacturer's instructions. cDNA was diluted with sterile water (1:2) and 2 μL were used for PCR or qPCR analysis.

Polymerase Chain Reaction

Polymerase chain reaction (PCR) was performed according to standard methods to test for pre-existing virus infections since bees were obtained from colonies which are subject to naturally occurring infections (*27, 43*). In brief, 2 μl cDNA template was combined with 10 pmol of each forward and reverse primers and amplified with ChoiceTaq polymerase according to the manufacturer's instructions using the following conditions: 95°C for 5min; 95°C for 30 s, 57°C for 30 s, 72°C for 30 s, 35 cycles, followed by final elongation at 72°C for 4 min. PCR products were assessed by gel electrophoresis (1.5% agarose with SYBR safe) and visualized using a Syngene U:Genius 3 gel imaging system (fig. S2). All products were previously verified by sequencing (*27*). Pathogen-specific PCR was used to detect DWV and SBV, as well as eight additional potential pre-existing virus infections and five non-viral pathogens on pooled samples of mock-infected bees for all experiments. Previous studies demonstrated the sensitivity and utility of pooled cDNA samples for pathogen testing [23].

Mock-infected honey bees from each of these experiments had preexisting infections of DWV, experiments 1-3 and 5 had preexisting SBV, and experiment 2 had preexisting IAPV, but were negative for all other pathogens examined including black queen cell virus, *Paenibacillus larvae*,

Lotmaria passim, *Nosema ceranae*, Lake Sinai viruses, Andrena-associated bee virus-1, chronic bee paralysis virus, Kashmir bee virus, *Melissococcus plutonius*, and acute bee paralysis virus (data S2). The quality of cDNA was assessed via amplification of the honey bee housekeeping gene, *rpl8*. To illustrate this result in a single figure, cDNA from mock-infected individual bee samples from all experiments was pooled by experiment (i.e. n=12 from experiment 1, n=12 from experiment 2, n=12 from experiment 3, n=6 from experiment 4, and n = 12 from experiment 5) and PCR was repeated. The products of pathogen-specific PCRs using pooled cDNA (S), positive (+), and negative control (-) templates were analyzed by agarose gel electrophoresis (fig. S2B).

Quantitative PCR

Quantitative PCR (qPCR) was used to analyze virus abundance and the relative abundance of select honey bee transcripts. All qPCR reactions were performed in triplicate with 2 µL of cDNA template. Each 20 µL reaction contained 1× ChoiceTaq Mastermix, 0.4 µM each forward and reverse primer, 1× SYBR Green (Life Technologies, Carlsbad, CA, USA), and 3 mM $MgCl_2$. A CFX Connect Real Time instrument (BioRad, Hercules, CA, USA) was used for the following thermo-profile: pre-incubation 95°C for 1min followed by 40 cycles of 95°C for 10 s, 58°C for 20 s, and 72°C for 15 s, with a final melt curve analysis at 65°C for 5 s to 95°C. To quantify viral copy numbers in the samples, plasmid standards (i.e., virus-specific qPCR amplicons cloned into the pGEM-T (Promega) vector and sequence verified) for each virus were used as templates, with concentrations ranging from 10^3-10^9 copies per reaction to create a linear standard curve. See data S3 for representative linear equations and primer efficiency calculations for each qPCR primer set. The host gene *Am rpl8* was amplified in triplicate for each sample for comparison and to assess cDNA quality (data S2). Reactions without template were used as negative controls. qPCR specificity was verified by melt point analysis, gel electrophoresis, and sequencing (*27*). The qPCR primers targeting DWV bind to both DWV-A (GenBank AY292384) and DWV-B (GenBank MN565036), as well as numerous other DWV sequences on NCBI including the consensus sequences of the DWV inoculum and naturally occurring DWV sequenced as part of this study. The qPCR primers amplify a region from 1165-1360 on DWV-A (GenBank AY292384). For each honey bee sample the starting quantity (SQ) for each well with cDNA template representing 80 ng total honey bee RNA was calculated based on the standard curve and subtracting the average SQ of the no template control reaction (i.e., <600 copies). Values below the limit of detection for qPCR (i.e., <1000 RNA copies / 2 µg RNA) were listed as 0, to retain conservative estimates. Virus abundance was reported as virus RNA copies, including genomes and transcripts, per 2 µg total RNA (i.e., per RT reaction) and ranged up to 9.9×10^9 virus RNA copies/2 µg RNA. Comparison of virus abundance levels using estimated copy numbers per 2 µg RNA allowed inclusion of values of 0; using the ΔΔCt method to calculate virus levels in bees with no detectable virus would inaccurately result in ‘missing’ data and consequently skew model interpretations. To estimate the virus abundance per individual honey bee, using an average total RNA yield of 140 µg per bee (data S1), the values reported per 2 µg RNA were multiplied by 70 (i.e., 140 µg total RNA/2 µg RNA) and were 3.6×10^5–2.6×10^{11} virus RNA copies per individual honey bee.

Relative host gene expression was calculated using the average ΔΔCt method where ΔCt was determined by subtracting *rpl8* Ct from the gene of interest Ct. Average ΔCt was calculated by the mean ΔCt of the buffer-injected bees, which harbored the lowest virus levels in each

experiment. The ΔΔCt was calculated by subtracting the average control ΔCt and fold change was determined by the equation $2^{-\Delta\Delta Ct}$ (data S1). Log_2 transformations were performed as necessary.

Statistics
Statistical analyses were performed in R 4.1.2. Log-transformations and non-parametric tests were performed as necessary, values of 1 were added before log transformations to retain data with values of 0.

Statistical analysis of pathogen abundance
Honey bees in this study were positive for DWV, SBV, and limited IAPV, therefore total pathogen abundance for each bee was determined as the sum of all viruses, normalized to 2 µg of RNA per bee. Both total and individual virus abundance data was unequally distributed, therefore log_{10} transformed values were utilized for statistical analyses. To account for values of 0, 1 was added to each raw number prior to log_{10} transformation. Low and high virus abundance were defined as less or greater than 10^6 virus RNA copies/2 µg RNA, respectively.

Linear relationship between virus abundance and flight distances
The relationship between flight distance, virus abundance, and other factors was assessed using linear mixed models from the lmer4 package (*87*). Assumptions for linear model analyses were verified by diagnostic plots and histograms. Various models evaluating individual honey bee weight, individual honey bee ID, colony ID, honey bee cage ID, day of individual bee frame-emergence, treatment group, and flight mill ID as random effects were considered with maximum likelihood (ML) when random effects varied or restricted maximum likelihood (REML) when fixed effects varied, in addition to Akaike information criterion corrected (AICc), and Bayesian information criterion (BIC) values (data S4). The best model with flight distance as the response variable, included virus abundance (log_{10} virus copies/2 µg RNA) as a fixed-response and mill ID and colony/experiment ID as random effects. The best-fit linear mixed model equations for DWV and total virus abundance can all be described as follows: [$y_{ij}=\beta_0+\beta_1 X_{1ij}+ \beta_2 X_{2ij}+\beta_3 X_{3ij}+z_1 u_i+ z_2 u_j +\varepsilon_{ij}$], where $y_{ij} = log_{10}(1+distance)$, $X_{1ij} = log_{10}$ (1+ DWV abundance), $X_{2ij} = log_{10}$(1+SBV abundance), $\beta_3 X_{3ij}$ = whole bee weight, $z_1 u_i$ = mill ID, and $z_2 u_j$ = colony/experiment. Model assumptions were assessed using the performance package in R. To predict values using the linear mixed model, input values were compared via the predict function in the stats package in R using average whole bee weight of 84.3 mg.

Figures
All figures were generated through Prism 9 software for windows using version 9.4.1, in RStudio using version R 4.1.2, in BioRender, and Adobe Illustrator.

Figure S1. Honey bee flight mill design and flight assays

(A) The overall design of the flight mills used in this study to quantify the flight distance of individual honey bees was based on previous models. Modifications include a magnet on the flight arm that is used to tether an individual bee to the mill, a flight arm capable of vertical movement, and automated flight data recording and a single board computer (i.e., Raspberry Pi) and a microSD card for data storage.
(B) Honey bees were either mock-infected or experimentally infected with DWV and flight behaviors were characterized at 72 hpi. Honey bees tethered to flight mills (1) flew and distance and time were recorded (Movie S1).
In addition, qualitative differences in behaviors and movements were observed in tethered bees, specifically (2) mock-infected bees were more active and exhibited more grooming behaviors (Movie S2) than (3) DWV-infected bees, which seemed more lethargic (Movie S3).

A

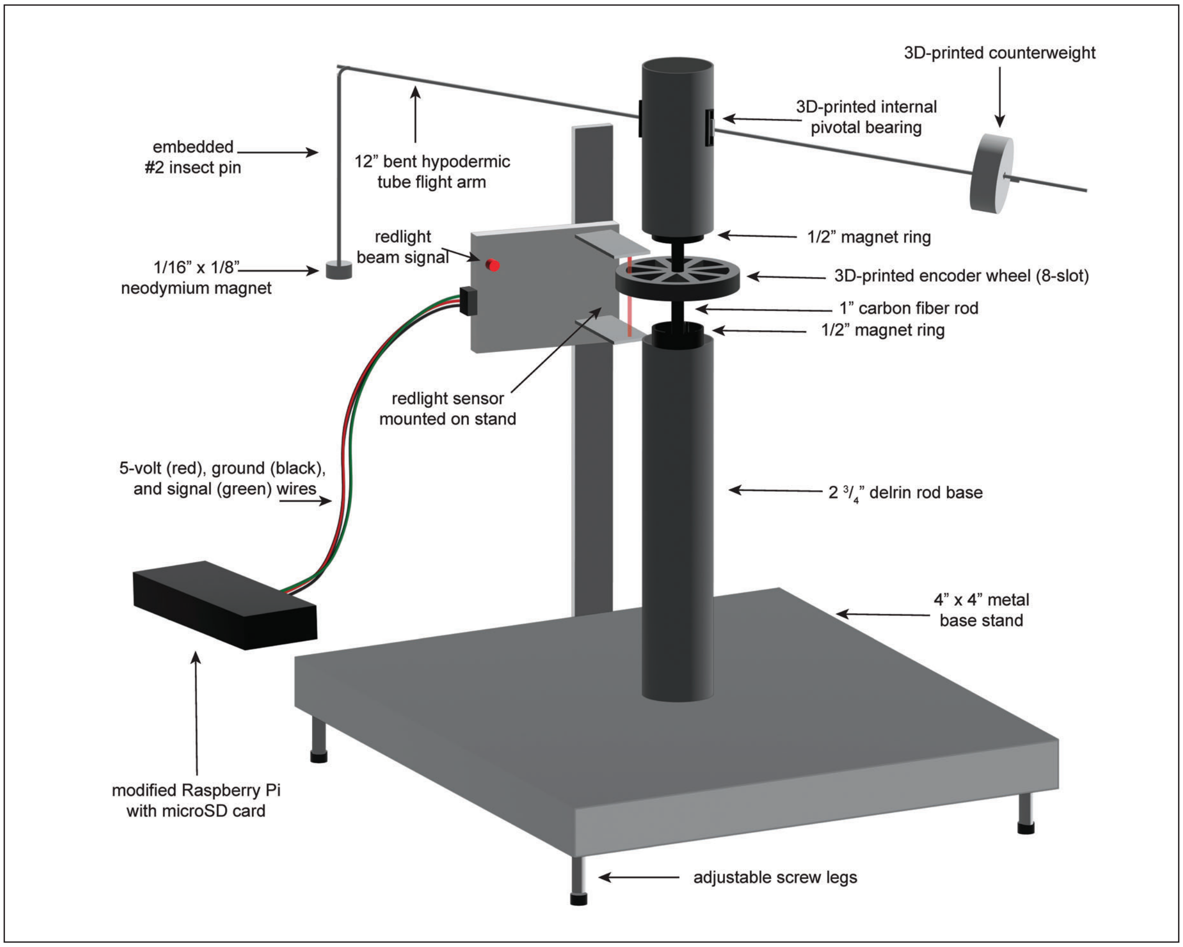

B

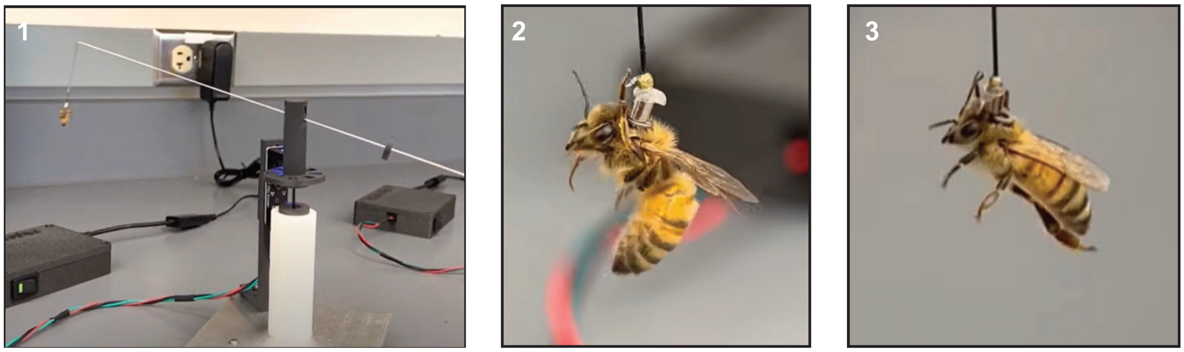

Figure S2. Pathogen testing of virus inoculum and honey bee samples

(A) Filtered virus inoculums DWV, SBV, and DWV+SBV (labeled A, B, or C, respectively) were tested for other common viruses including acute bee paralysis virus (ABPV), Apis mellifera filamentous virus (AmFV), Andrena bee-associated virus-1 (AnBV), black queen cell virus (BQCV), chronic bee paralysis virus (CBPV), deformed wing virus (DWV), Israeli acute paralysis virus (IAPV), Kashmir bee virus (KBV), Lake Sinai viruses 1-4 (LSV), and sacbrood virus (SBV) using virus-specific polymerase chain reaction (PCR) and analyzed by gel electrophoresis; positive (+) and negative (-) controls. (B) Pathogen diagnostic PCR was performed using pooled mock-infected honey bee cDNA from experiments 1-5 (labeled 1-5) as template determined that bees had preexisting DWV (experiments 1-5), SBV (experiments 1-3, and 5), and IAPV (experiment 4). When preexisting infections were detected, quantitative-PCR (qPCR) was used to determine virus abundance in individual bee samples (Table S1). All experiments were negative for all non-viral pathogens including *Ascosphaera apis* (*Aa.*), *Lotmaria passim* (*Lp.*), *Melisococcus plutonius* (*Mp.*), *Nosema ceranae* (*Nc.*), and *Paenibacillus larvae* (*Pl.*); cDNA quality was assessed via amplification of the honey bee housekeeping gene, *rpl8*. Although no positive control was available for *A. apis* or *P. larvae*, the primers were utilized successfully in previous studies (15, 16).

To illustrate this result in a single figure, cDNA from mock-infected individual bee samples from all experiments was pooled by experiment (i.e. n=12 from experiment 1, n=12 from experiment 2, n=12 from experiment 3, n=6 from experiment 4, and n = 12 from experiment 5) and PCR was repeated. The products of pathogen-specific PCRs using pooled cDNA (S), positive (+), and negative control (-) templates were analyzed by agarose gel electrophoresis.

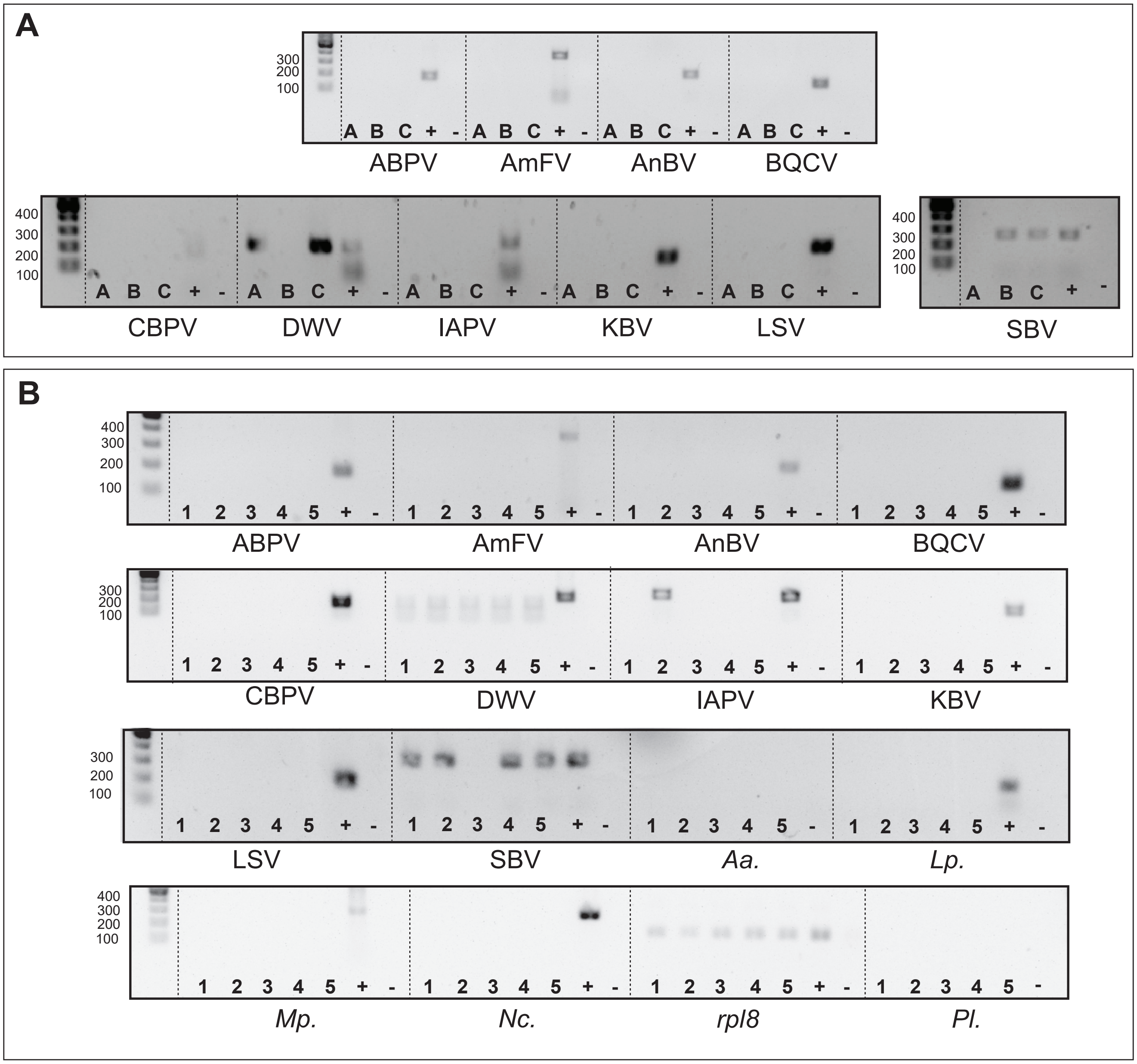

Figure S3. RNA yield and virus quantification

(A) The amount of RNA isolated from honey bee head, thorax, and abdomen samples was proportional to segment weight. Honey bees were dissected into three body segments (i.e., n=15, head, thorax, abdomen) and weighed, then RNA was isolated from each sample. RNA yield was evaluated for each sample and was consistent across segments (ANOVA, F = 0.36, *p*=0.78). RNA isolation was most consistent from abdomen samples, whereas low RNA yields were obtained from three head samples and one thorax sample (i.e., <100 ng RNA total, not represented on graph); overnight RNA precipitation enhanced head sample yield. (B) RNA yield slightly varied for each individual bee segment (ID 1-15) and dots as indicators of head, thorax, abdomen, and whole bee yields. (C) To determine if DWV quantification from honey bee abdomen samples is representative of levels in whole bees, qPCR was used to determine DWV abundance in each honey bee segment. In general, data from head and thorax samples underestimated virus abundance (i.e., three head samples and one thorax sample were DWV-negative). Virus abundance data obtained from abdomen samples were most consistent (i.e., produced no false negatives). DWV levels in abdomen samples were greater than whole bee estimates, as head and thorax data lowered the average (Mann-Whitney, *U*=50, *p*=0.008). (D) DWV abundance was examined in each individual bee segment (ID 1-15). In this group, the two honey bees experimentally infected with DWV (ID 14 and 15) had higher DWV and SBV loads than mock-infected controls (ID 1-13). (E) Quantification of SBV abundance in each honey bee segment revealed that data from abdomens was consistent with whole bee averages (Mann-Whitney, *U*=78, *p*=0.16), whereas three thorax samples falsely indicated that those bees were SBV-negative. (F) SBV abundance was examined in each individual bee segment (ID 1-15) and highlighted inconsistency in SBV data obtained from thorax samples (i.e., three SBV-negative samples (ID 1, 4, and 11) while the corresponding head and abdomen samples were SBV positive).

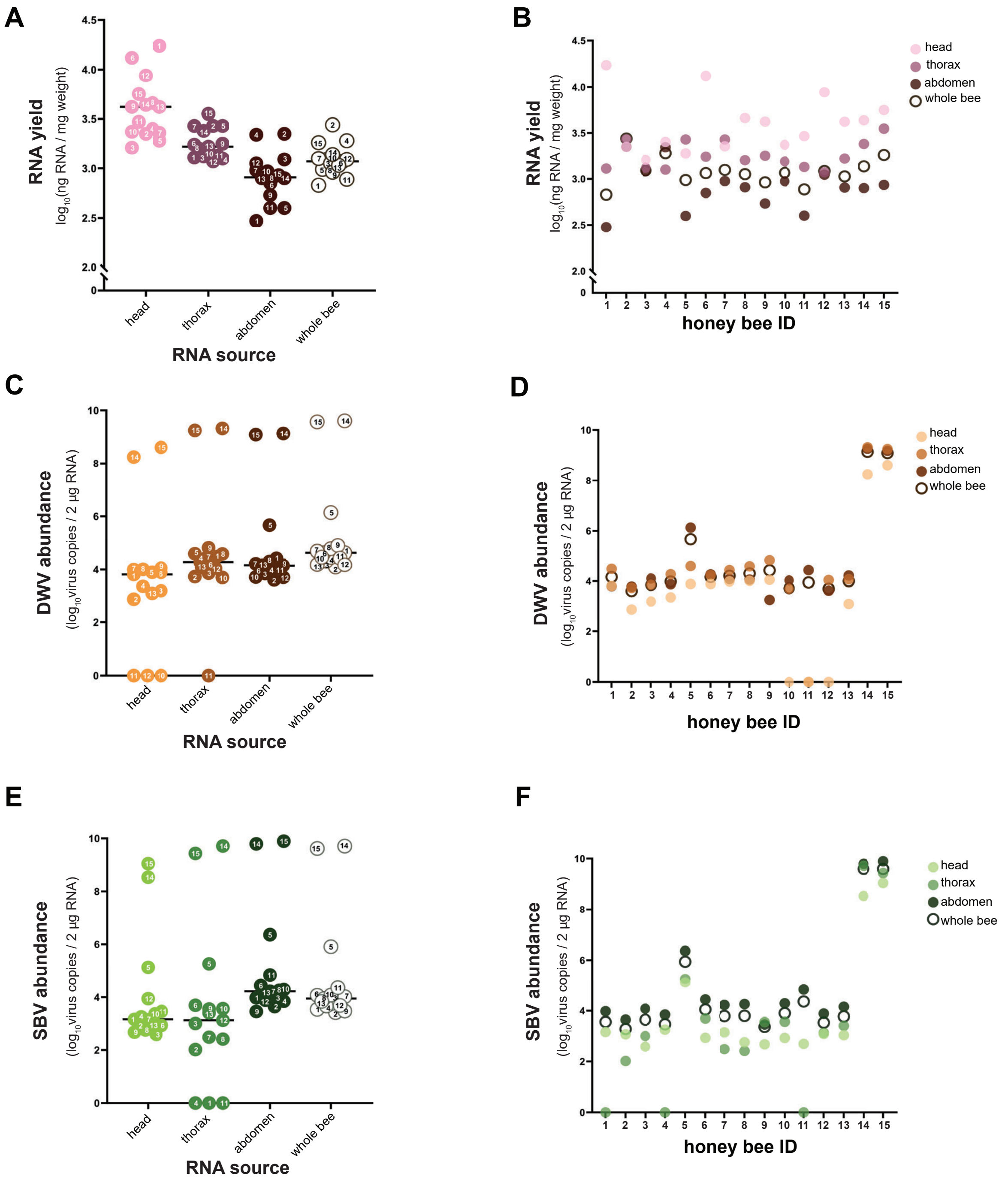

Figure S4. Honey bees with Israeli acute paralysis virus (IAPV) infections had impaired flight
(A) A small number of honey bees from experiment two had detectable, preexisting infections of Israeli acute paralysis virus (IAPV) (geometric mean = 4 x 10^5 IAPV RNA copies / 2µg RNA). (B) To compare flight capability, the flight distance ($\log_{10}$ meters) was compared between mock infected, IAPV-negative bees, and IAPV-positive bees from the same experiment with the IAPV positive bees. IAPV-postive bees flew 71% shorter distances than IAPV-negative honey bees (Mann-Whitney, $U = 52$, $p = 0.02$). (C) Additionally, IAPV-infected bees flew at slower speeds (Mann-Whitney, $U = 58$, $p = 0.04$). (D) To estimate the frequency that IAPV infected bees stopped to engage in grooming behaviors, flight stop count was calculated. IAPV-infected honey bees stopped 63% less often than IAPV-negative bees in the same experiment (Mann-Whitney, $U = 52$, $p = 0.03$).

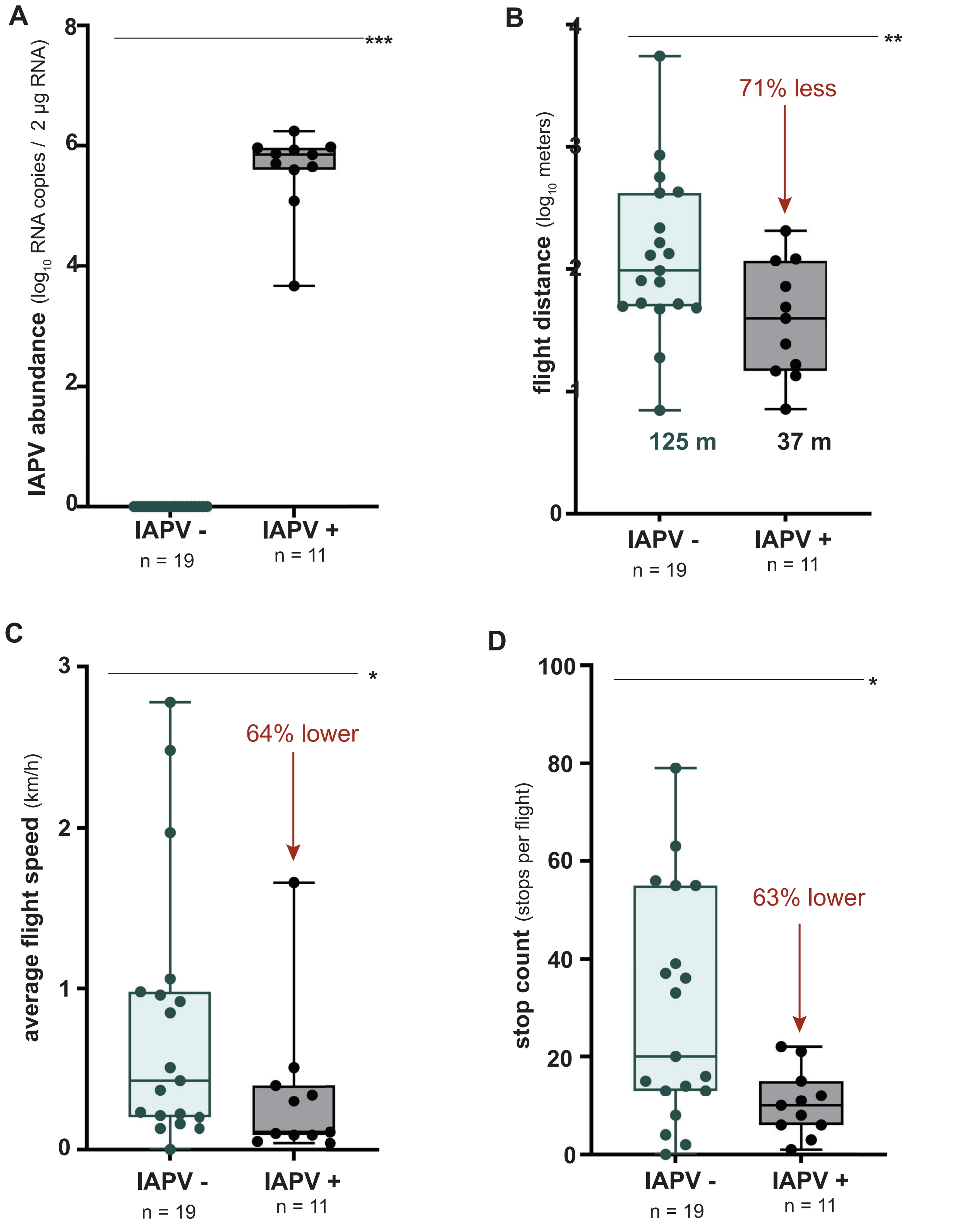

Figure S5. Whole bee weight was negatively associated with flight distance
The relationship between whole bee weight (mg) and flight distance (log_{10} meters) was statistically evaluated using a linear mixed model with virus abundance (i.e., DWV and SBV) as fixed variables and experiment, treatment, and flight mill as random effects (Figure 1, Table S4). This indicated a negative trend between whole bee weight and flight distance (regression coefficient = -0.01, p <0.01). Each point in the effect plot represents data from one individual bee after accounting for fixed and random effects of the best-fit model. The thin, curved line represents the observed relationship between whole bee weight and flight distance, the straight line is the model prediction, the shaded band represents the 95% confidence interval of prediction estimates.

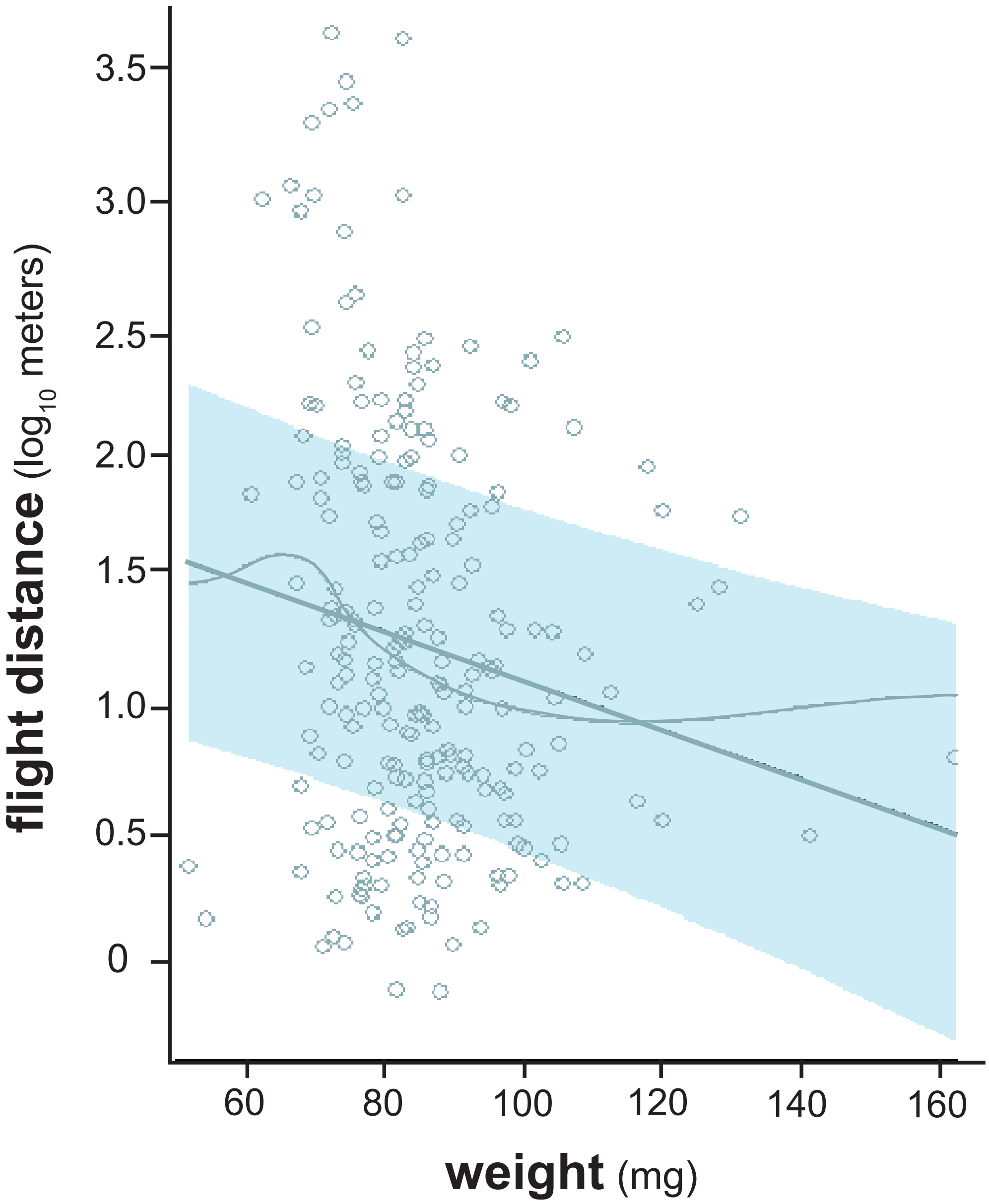

Figure S6. Highly DWV infected honey bees stopped less frequently to engage in grooming behaviors

While evaluating flight performance in mock-infected and virus-infected honey bees, we noticed that bees stopped and exhibited grooming behaviors in effort to detach from flight arms. Counterintuitively, bees seemed to expend a lot of energy during these stops (Figure S1) and therefore, flight stop count may serve as another indicator of energy expenditure. Analyses of the frequency that honey bees stopped during flight (i.e., $\log_{10}$ (total number of stops > 1.5 seconds per total flight)) was evaluated in relation to virus abundance and revealed that honey bees with greater DWV abundance levels stopped less than bees harboring lower DWV levels or no DWV levels (i.e., averaged 25 stops, 11 stops and 10 stops, respectively); (Mann-Whitney comparison of no DWV to low DWV, $U = 579$, $p<0.0001$), (Mann-Whitney comparison of low DWV to high DWV, $U = 1191$, $p <0.0001$), (Mann-Whitney comparison of low DWV to high DWV, $U = 2850$, $p = 0.02$).

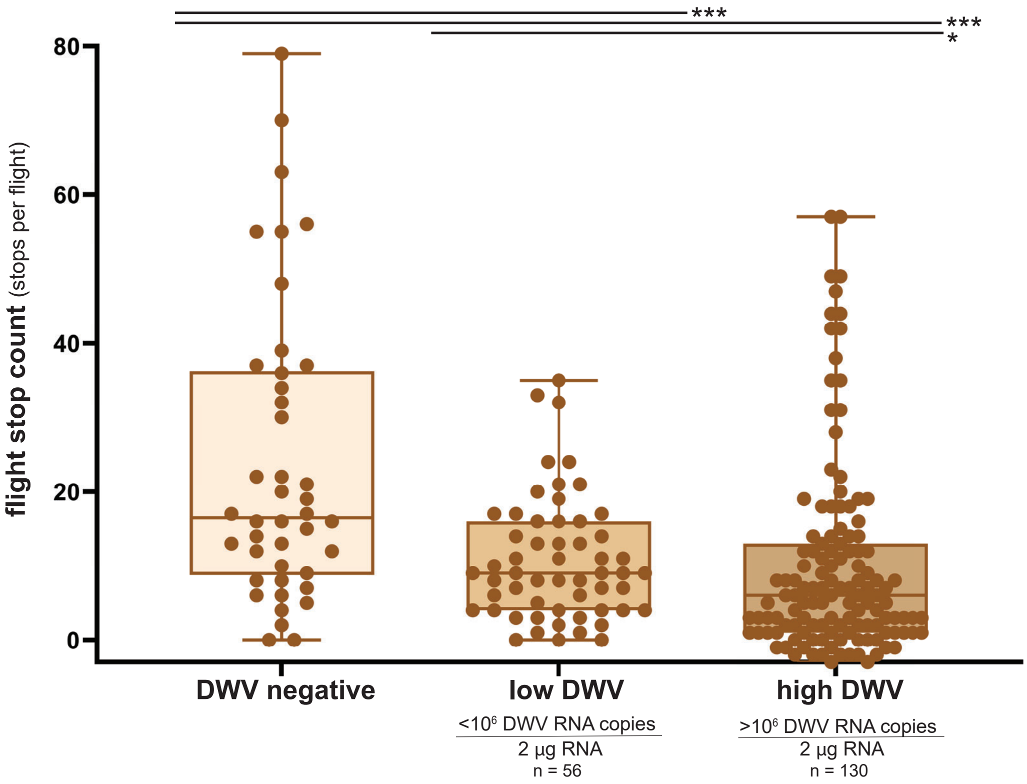

Figure S7. Honey bees with high deformed wing virus (DWV) loads expressed greater levels of a small heat shock protein (*pl2*)

To investigate relationships between DWV abundance and *protein lethal essential for life-like(2)* (*pl2*) expression, *pl2* transcript abundance was normalized to housekeeping gene, *ribosomal protein l8* (*rpl8*) and fold change was calculated using ΔΔCt method relative to the average expression in mock-infected bees for each experiment. DWV abundance was categorized into low DWV level (i.e., <10^6 virus RNA copies / 2µg RNA) or high DWV level (i.e., >10^6 virus RNA copies / 2µg RNA). The median level of *pl2* expression was greater in honey bees with high DWV loads in two of the four experiments analyzed (i.e., in experiments 2 and 5, Mann-Whitney, U = 38, p = 0.005; U = 34, p < 0.001, respectively).

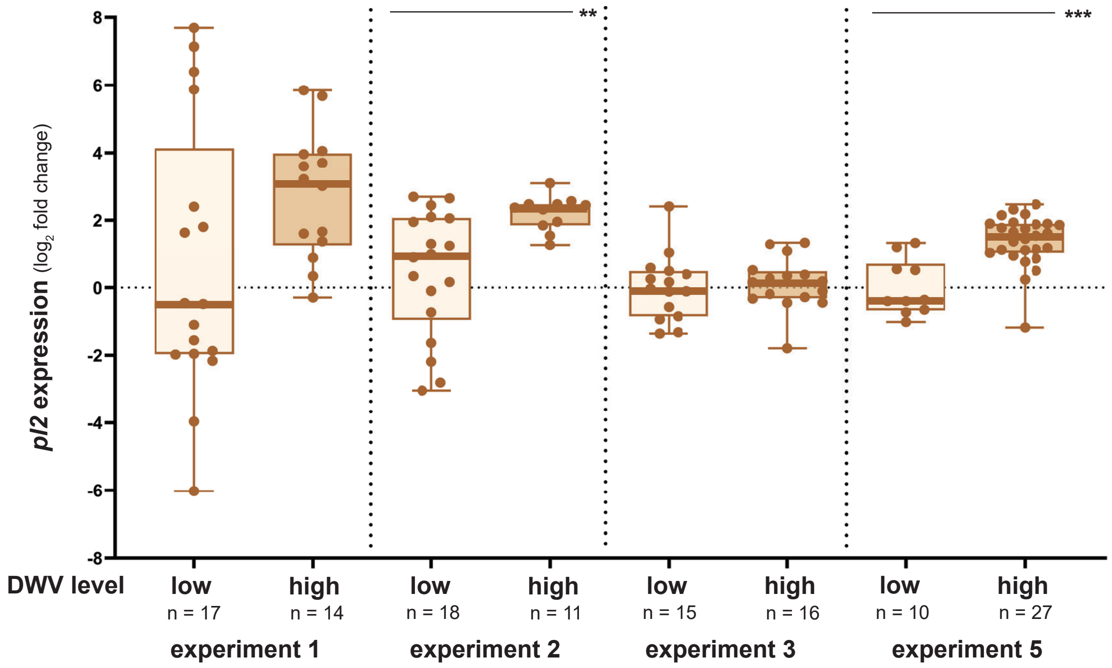

Figure S8. Highly virus-infected bees had greater expression of *heat shock protein 90* (*hsp90*)
To investigate relationships between total virus abundance (i.e., sum of DWV and SBV) and *heat shock protein 90* (*hsp90*) expression, *hsp90* transcript abundance was normalized to housekeeping gene, *ribosomal protein l8* (*rpl8*) and fold change was calculated using ΔΔCt method relative to the average expression in mock-infected bees for each experiment. Total virus abundance was categorized into low total virus level (i.e., $<10^6$ virus RNA copies / 2µg RNA) or high total virus level (i.e., $>10^6$ virus RNA copies / 2µg RNA). In each of the three experiments *hsp90* expression was quantified, *hsp90* expression was greater in bees with high virus loads (Mann-Whitney, U = 30, p < 0.001; U = 0, p < 0.001; U = 47, p = 0.003, respectively).

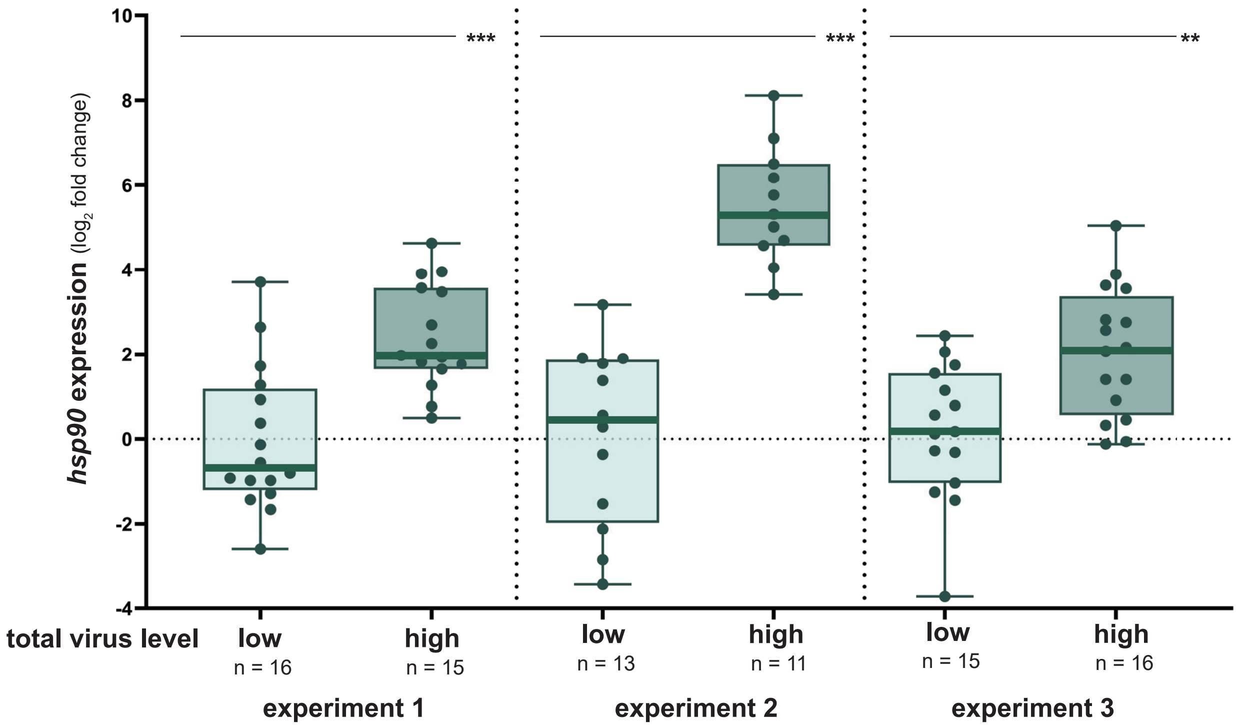

Figure S9. Honey bees infected with sacbrood virus (SBV) had greater expression of an octopamine receptor (*Oß-2R*)

The relationship between SBV levels and octopamine receptor (*Oß-2R*), expression was evaluated in bees from each experiment. SBV was detected for individual bees via qPCR and denoted as SBV negative or SBV positive (neg. or pos., respectively). Transcript abundance was normalized to housekeeping gene, *ribosomal protein l8* (*rpl8*) and fold change was calculated using ΔΔCt method relative to the average expression in SBV negative bees from each experiment. Honey bees with detectable SBV had greater Oß2-R expression in experiments 1, 4, and 5 (Mann-Whitney, 3.3x greater, U = 5, p = 0.003; 6.5x greater, U = 82, p = 0.03; 1.8x greater, U = 601, p <0.001, respectively). Experiment 3 had no preexisting or experimentally introduced SBV infections and the number of bees in experiment 2 that was assessed was insufficient for in experiment statistical analysis.

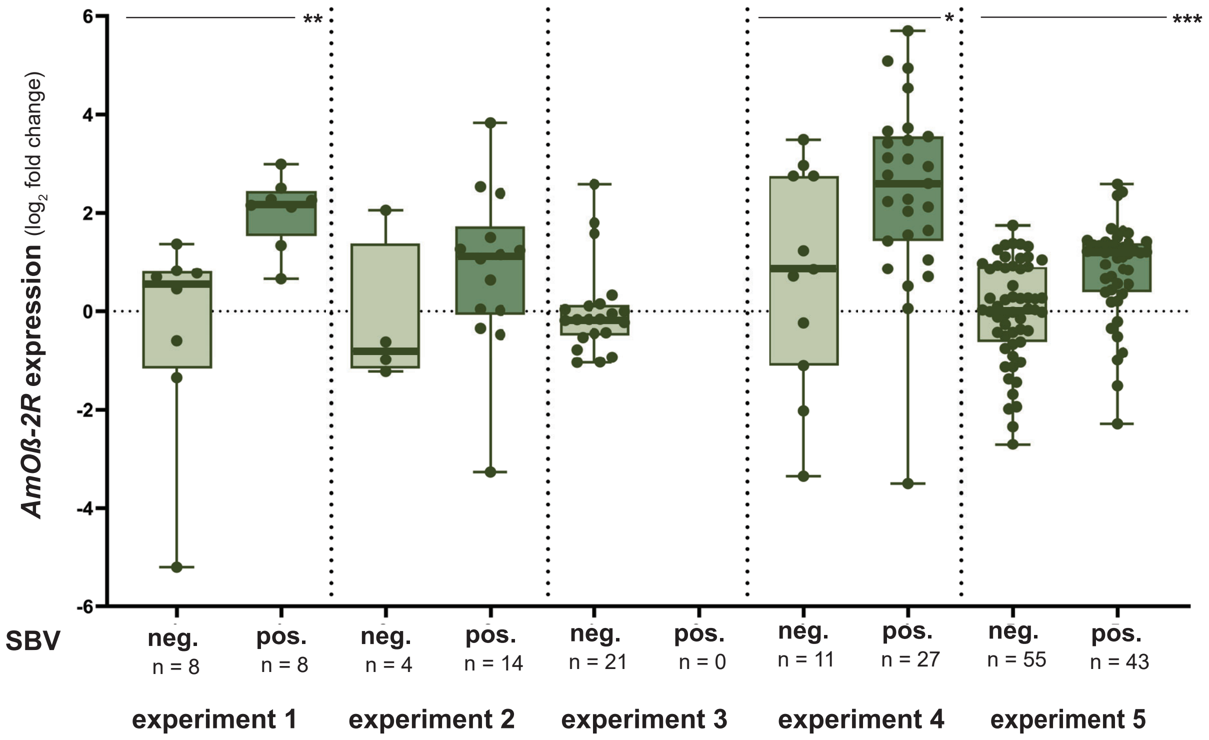

Figure S10. Honey bee sucrose consumption does not scale with flight performance
To examine the potential relationships between honey bee sucrose consumption and virus infection, we measured sucrose consumption in mock infected (buffer injected) honey bees which harbored less virus and flew greater distances, and virus-inoculated honey bees (Table S6). (A) The amount of sucrose patty consumed by honey bees in experiments 3, 4, and 5, is represented as the average sucrose consumed by cohoused honey bees (each point on the graph; avg./mg/bee/day). Virus infected honey bees consumed similar sucrose levels, and mock infected bees did not consume more sucrose than virus-infected bees. (B) In an additional experiment, mock infected bees fed 50% sucrose syrup consumed 46 μL less sucrose syrup than DWV and SBV co-infected bees.

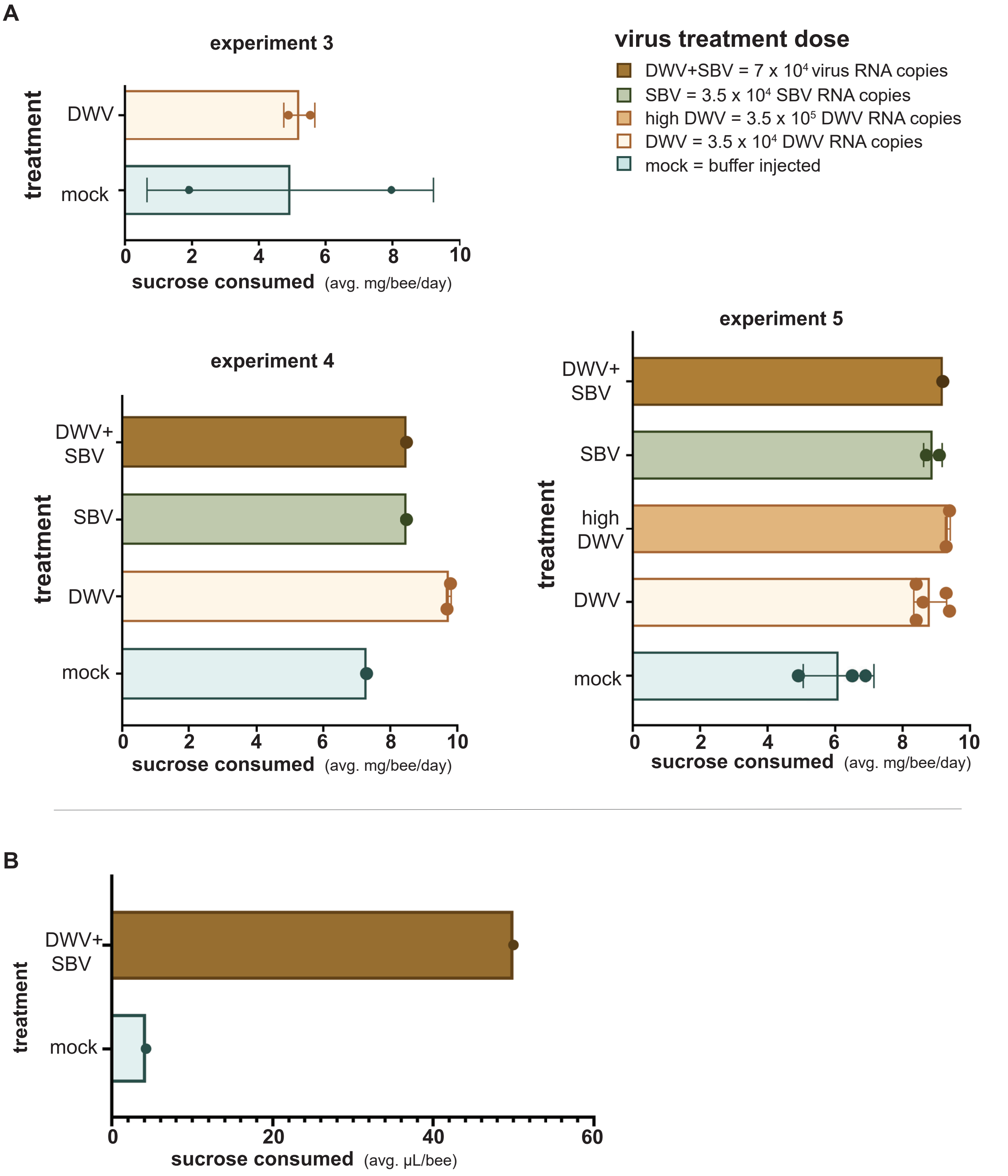

Movie S1.

Honey bees were either mock-infected or experimentally infected with DWV and flight behaviors were characterized at 72 hpi. Honey bees tethered to flight mills flew and distance and time were recorded.

Movie S2.

Qualtitative differences in behaviors and movements were observed in tethered bees, specifically, mock-infected bees were more active and exhibited more grooming behaviors.

Movie S3.

Tethered, DWV-infected bees were more lethargic and exhibited fewer grooming behaviors.

Data S1. (separate file)

Flight distance, virus abundance, and gene expression data for all individual honey bees samples, including all treatment groups and experiments.

Data S2. (separate file)

Primers used in this study; to identify and quantify pathogen abundance and honey bee gene expression via endpoint PCR and qPCR, we utilized 26 pairs of sequence specific primers.

Data S3. (separate file)

Quantification of virus abundance in samples was assessed using the standard curves generated from known quantities of plasmid standards as template DNA on each qPCR plate with samples.

Data S4. (separate file)

Linear mixed model selection data used for selecting best-fit models.

Data S5. (separate file)

Honey bee forewing lengths and whole body weight data.

Data S6. (separate file)

Worksheet to estimate flight distance reduction at the colony level due to inapparent DWV infection.

Data S7. (separate file)

Virus quantification and sucrose consumption measurements.

Data S8. (separate file)

Template including macros to calculate flight duration, active flight, flight stop frequency, flight distance, and flight speeds.

Data S9. (separate file)

Python script for auto calculation of flight distance and active flight duration from the flight mill generated csv files.

Data S10. (separate file)

Honey bee RNA yield and virus abundance by bee segment (i.e., head, thorax, abdomen, and whole bee).

REFERENCES AND NOTES

1. J. Hilaire, S. Tindale, G. Jones, G. Pingarron-Cardenas, K. Bačnik, M. Ojo, L. J. Frewer, Risk perception associated with an emerging agri-food risk in Europe: Plant viruses in agriculture. *Agric. Food Secur.* **11**, 21 (2022).

2. E. Harvey, E. C. Holmes, Diversity and evolution of the animal virome. *Nat. Rev. Microbiol.* **20**, 321–334 (2022).

3. S. Asgari, K. N. Johnson, *Insect virology* (Norfolk, 2010).

4. A. Mussabekova, L. Daeffler, J. L. Imler, Innate and intrinsic antiviral immunity in Drosophila. *Cell. Mol. Life Sci.* **74**, 2039–2054 (2017).

5. G. H. Samuel, Z. N. Adelman, K. M. Myles, Antiviral immunity and virus-mediated antagonism in disease vector mosquitoes. *Trends Microbiol.* **26**, 447–461 (2018).

6. J. T. Marques, C. Meignin, J. L. Imler, An evolutionary perspective to innate antiviral immunity in animals. *Cell Rep.* **43**, 114678 (2024).

7. M. L. Winston, *The Biology of the Honey Bee* (Harvard Univ. Press, 1991).

8. Food and Agriculture Organization of the United Nations (FAO), 2023, FAOSTAT Production: Crops and livestock products; Accessed on June 1, 2025; https://www.fao.org/faostat/en/#data/QCL.

9. F. J. Balvino-Olvera, J. A. Lobo, M. J. Aguilar-Aguilar, G. Ruiz-Guzmán, A. González-Rodríguez, I. Ruiz-Mercado, A. Ghilardi, M. del Coro Arizmendi, M. Quesada, Long-term spatiotemporal patterns in the number of colonies and honey production in Mexico. *Sci. Rep.* **13**, 1017 (2023).

10. D. vanEngelsdorp, D. Caron, J. Hayes, R. Underwood, M. Henson, K. Rennich, A. Spleen, M. Andree, R. Snyder, K. Lee, K. Roccasecca, M. Wilson, J. Wilkes, E. Lengerich, J. Pettis, A national survey of managed honey bee 2010–11 winter colony losses in the USA: Results from the Bee Informed Partnership. *J. Apic. Res.* **51**, 115–124 (2012).

11. D. vanEngelsdorp, J. Hayes, R. M. Underwood, J. Pettis, A survey of honey bee colony losses in the U.S., fall 2007 to spring 2008. *PLOS ONE* **3**, e4071 (2008).

12. S. Bruckner, M. Wilson, D. Aurell, K. Rennich, D. vanEngelsdorp, N. Steinhauer, G. R. Williams, A national survey of managed honey bee colony losses in the USA: Results from the Bee Informed Partnership for 2017–18, 2018–19, and 2019–20. *J. Apic. Res.* **62**, 429–443 (2023).

13. R. van der Zee, L. Pisa, S. Andonov, R. Brodschneider, J.-D. Charriere, R. Chlebo, M. F. Coffey, K. Crailsheim, B. Dahle, A. Gajda, A. Gray, M. M. Drazic, M. Higes, L. Kauko, A. Kence, M. Kence, N. Kezic, H. Kiprijanovska, J. Kralj, P. Kristiansen, R. M. Hernandez, F. Mutinelli, B. K. Nguyen, C. Otten, A. Özkırım, S. F. Pernal, M. Peterson, G. Ramsay, V. Santrac, V. Soroker, G. Topolska, A. Uzunov, F. Vejsnaes, S. Wei, S. Wilkins, Managed honey bee colony losses in Canada, China, Europe, Israel and Turkey, for the winters of 2008–9 and 2009–10. *J. Apic. Res.* **51**, 100–114 (2015).

14. D. Aurell, S. Bruckner, M. Wilson, N. Steinhauer, G. R. Williams, A national survey of managed honey bee colony losses in the USA: Results from the Bee Informed Partnership for 2020–21 and 2021–22. *J. Apic. Res.* **63**, 1–14 (2024).

15. Z. Lamas. (Project Apis m., 2025).

16. K. S. Traynor, K. Rennich, E. Forsgren, R. Rose, J. Pettis, G. Kunkel, S. Madella, J. Evans, D. Lopez, D. vanEngelsdorp, Multiyear survey targeting disease incidence in US honey bees. *Apidologie* **47**, 325–347 (2016).

17. D. vanEngelsdorp, J. Hayes, R. M. Underwood, J. S. Pettis, A survey of honey bee colony losses in the United States, fall 2008 to spring 2009. *J. Apic. Res.* **49**, 7–14 (2010).

18. E. Genersch, W. von der Ohe, H. Kaatz, A. Schroeder, C. Otten, R. Buchler, S. Berg, W. Ritter, W. Muhlen, S. Gisder, M. Meixner, G. Liebig, P. Rosenkranz, The German bee monitoring project: A long term study to understand periodically high winter losses of honey bee colonies. *Apidologie* **41**, 332–352 (2010).

19. D. Goulson, E. Nicholls, C. Botias, E. L. Rotheray, Bee declines driven by combined stress from parasites, pesticides, and lack of flowers. *Science* **347**, 1255957 (2015).

20. F. Nazzi, F. Pennacchio, Disentangling multiple interactions in the hive ecosystem. *Trends Parasitol.* **30**, 556–561 (2014).

21. Y. P. Chen, J. S. Pettis, M. Corona, W. P. Chen, C. J. Li, M. Spivak, P. K. Visscher, G. DeGrandi-Hoffman, H. Boncristiani, Y. Zhao, D. vanEngelsdorp, K. Delaplane, L. Solter, F. Drummond, M. Kramer, W. I. Lipkin, G. Palacios, M. C. Hamilton, B. Smith, S. K. Huang, H. Q. Zheng, J. L. Li, X. Zhang, A. F. Zhou, L. Y. Wu, J. Z. Zhou, M.-L. Lee, E. W. Teixeira, Z. G. Li, J. D. Evans, Israeli acute paralysis virus: Epidemiology, pathogenesis and implications for honey bee health. *Plos Pathog.* **10**, e1004261 (2014).

22. Y. P. Chen, R. Siede, "Honey bee viruses" in *Advances in Virus Research* (Elsevier, 2007), vol. 70, pp. 33–80.

23. C. M. Grozinger, M. L. Flenniken, Bee viruses: Ecology, pathogenicity, and impacts. *Annu. Rev. Entomol.* **64**, 205–226 (2019).

24. R. Wei, L. Cao, Y. Feng, Y. Chen, G. Chen, H. Zheng, Sacbrood virus: A growing threat to honeybees and wild pollinators. *Viruses* **14**, (2022).

25. D. Annoscia, S. P. Brown, G. Di Prisco, E. De Paoli, S. Del Fabbro, D. Frizzera, V. Zanni, D. A. Galbraith, E. Caprio, C. M. Grozinger, F. Pennacchio, F. Nazzi, Haemolymph removal by Varroa mite destabilizes the dynamical interaction between immune effectors and virus in bees, as predicted by Volterra's model. *Proc. Biol. Sci.* **286**, 20190331 (2019).

26. F. Nazzi, S. P. Brown, D. Annoscia, F. Del Piccolo, G. Di Prisco, P. Varricchio, G. Della Vedova, F. Cattonaro, E. Caprio, F. Pennacchio, Synergistic parasite-pathogen interactions mediated by host immunity can drive the collapse of honeybee colonies. *Plos Pathog.* **8**, e1002735 (2012).

27. C. Faurot-Daniels, W. Glenny, K. F. Daughenbaugh, A. J. McMenamin, L. A. Burkle, M. L. Flenniken, Longitudinal monitoring of honey bee colonies reveals dynamic nature of virus

abundance and indicates a negative impact of Lake Sinai virus 2 on colony health. *PLOS ONE* **15**, e0237544 (2020).

28. W. Glenny, I. Cavigli, K. F. Daughenbaugh, R. Radford, S. E. Kegley, M. L. Flenniken, Honey bee (*Apis mellifera*) colony health and pathogen composition in migratory beekeeping operations involved in California almond pollination. *PLOS ONE* **12**, e0182814 (2017).

29. C. Runckel, M. L. Flenniken, J. C. Engel, J. G. Ruby, D. Ganem, R. Andino, J. L. DeRisi, Temporal analysis of the honey bee microbiome reveals four novel viruses and seasonal prevalence of known viruses, Nosema, and Crithidia. *PLOS ONE* **6**, e20656 (2011).

30. P. D'Alvise, V. Seeburger, K. Gihring, M. Kieboom, M. Hasselmann, Seasonal dynamics and co-occurrence patterns of honey bee pathogens revealed by high-throughput RT-qPCR analysis. *Ecol. Evol.* **9**, 10241–10252 (2019).

31. T. Wells, S. Wolf, E. Nicholls, H. Groll, K. S. Lim, S. J. Clark, J. Swain, J. L. Osborne, A. J. Haughton, Flight performance of actively foraging honey bees is reduced by a common pathogen. *Environ. Microbiol. Rep.* **8**, 728–737 (2016).

32. N. Mockel, S. Gisder, E. Genersch, Horizontal transmission of deformed wing virus: Pathological consequences in adult bees (*Apis mellifera*) depend on the transmission route. *J. Gen. Virol.* **92**, 370–377 (2011).

33. J. R. de Miranda, E. Genersch, Deformed wing virus. *J. Invertebr. Pathol.* **103**, S48–S61 (2010).

34. E. Genersch, M. Aubert, Emerging and re-emerging viruses of the honey bee (*Apis mellifera L.*). *Vet. Res.* **41**, 54 (2010).

35. S. Tosi, G. Burgio, J. C. Nieh, A common neonicotinoid pesticide, thiamethoxam, impairs honey bee flight ability. *Sci. Rep.* **7**, 1201 (2017).

36. R. Scheiner, C. I. Abramson, R. Brodschneider, K. Crailsheim, W. M. Farina, S. Fuchs, B. Grunewald, S. Hahshold, M. Karrer, G. Koeniger, N. Koeniger, R. Menzel, S. Mujagic, G.

Radspieler, T. Schmickl, C. Schneider, A. J. Siegel, M. Szopek, R. Thenius, Standard methods for behavioural studies of Apis mellifera. *J. Apicult. Res.* **52**, 1–58 (2013).

37. L. J. Blanken, F. van Langevelde, C. van Dooremalen, Interaction between Varroa destructor and imidacloprid reduces flight capacity of honeybees. *P R Soc B* **282**, 20151738 (2015).

38. R. Brodschneider, E. Omar, K. Crailsheim, Flight performance of pollen starved honey bees and incomplete compensation through ingestion after early life pollen deprivation. *Front. Physiol.* **13**, 1004150 (2022).

39. J. Gao, S. Ma, X. Wang, Y. Yang, Q. Luo, X. Wang, F. Liu, Q. Wang, Z. Fu, Q. Diao, P. Dai, Tropilaelaps mercedesae parasitism changes behavior and gene expression in honey bee workers. *Plos Pathog* **17**, e1009684 (2021).

40. K. Benaets, A. Van Geystelen, D. Cardoen, L. De Smet, D. C. de Graaf, L. Schoofs, M. H. Larmuseau, L. E. Brettell, S. J. Martin, T. Wenseleers, Covert deformed wing virus infections have long-term deleterious effects on honeybee foraging and survival. *Proc. Biol. Sci.* **284**, 20162149 (2017).

41. C. Alaux, D. Crauser, M. Pioz, C. Saulnier, Y. Le Conte, Parasitic and immune modulation of flight activity in honey bees tracked with optical counters. *J. Exp. Biol.* **217**, 3416–3424 (2014).

42. J. Carrillo-Tripp, A. G. Dolezal, M. J. Goblirsch, W. A. Miller, A. L. Toth, B. C. Bonning, In vivo and in vitro infection dynamics of honey bee viruses. *Sci. Rep.* **6**, 22265 (2016).

43. L. M. Brutscher, K. F. Daughenbaugh, M. L. Flenniken, Virus and dsRNA-triggered transcriptional responses reveal key components of honey bee antiviral defense. *Sci. Rep.* **7**, 6448 (2017).

44. J. R. de Miranda, L. Bailey, B. V. Ball, P. Blanchard, G. E. Budge, N. Chejanovsky, Y.-P. Chen, L. Gauthier, E. Genersch, D. C. de Graaf, M. Ribière, E. Ryabov, L. De Smet, J. J. M. van der Steen, Standard methods for virus research in *Apis mellifera*. *J. Apic. Res.* **52**, 1–56 (2013).

45. K. F. Daughenbaugh, M. Martin, L. M. Brutscher, I. Cavigli, E. Garcia, M. Lavin, M. L. Flenniken, Honey bee infecting Lake Sinai viruses. *Viruses* **7**, 3285–3309 (2015).

46. A. J. McMenamin, K. F. Daughenbaugh, M. L. Flenniken, The heat shock response in the Western honey bee (Apis mellifera) is antiviral. *Viruses* **12**, 245 (2020).

47. E. V. Ryabov, J. M. Fannon, J. D. Moore, G. R. Wood, D. J. Evans, The Iflaviruses Sacbrood virus and deformed wing virus evoke different transcriptional responses in the honeybee which may facilitate their horizontal or vertical transmission. *PeerJ* **4**, e1591 (2016).

48. S. Kaya-Zeeb, S. Delac, L. Wolf, A. L. Marante, O. Scherf-Clavel, M. Thamm, Robustness of the honeybee neuro-muscular octopaminergic system in the face of cold stress. *Front. Physiol.* **13**, 1002740 (2022).

49. S. Kaya-Zeeb, L. Engelmayer, M. Straßburger, J. Bayer, H. Bähre, R. Seifert, O. Scherf-Clavel, M. Thamm, Octopamine drives honeybee thermogenesis. *eLife* **11**, e74334 (2022).

50. W. Blenau, J. A. Wilms, S. Balfanz, A. Baumann, AmOctα2R: Functional characterization of a honeybee octopamine receptor inhibiting adenylyl cyclase activity. *Int. J. Mol. Sci.* **21**, 9334 (2020).

51. P. Sassone-Corsi, The cyclic AMP pathway. *Cold Spring Harb. Perspect. Biol.* **4**, a011148 (2012).

52. B. J. Hoye, V. J. Munster, N. Huig, P. de Vries, K. Oosterbeek, W. Tijsen, M. Klaassen, R. A. M. Fouchier, J. A. van Gils, Hampered performance of migratory swans: Intra- and inter-seasonal effects of avian influenza virus. *Integr. Comp. Biol.* **56**, 317–329 (2016).

53. T. J. O'Shea, P. M. Cryan, A. A. Cunningham, A. R. Fooks, D. T. Hayman, A. D. Luis, A. J. Peel, R. K. Plowright, J. L. Wood, Bat flight and zoonotic viruses. *Emerg. Infect. Dis.* **20**, 741–745 (2014).

54. C. M. Newman, T. K. Anderson, T. L. Goldberg, Decreased flight activity in Culex pipiens (Diptera: Culicidae) naturally infected with Culex flavivirus. *J. Med. Entomol.* **53**, 233–236 (2016).

55. E. A. Capaldi, A. D. Smith, J. L. Osborne, S. E. Fahrbach, S. M. Farris, D. R. Reynolds, A. S. Edwards, A. Martin, G. E. Robinson, G. M. Poppy, J. R. Riley, Ontogeny of orientation flight in the honeybee revealed by harmonic radar. *Nature* **403**, 537–540 (2000).

56. J. T. Vance, J. B. Williams, M. M. Elekonich, S. P. Roberts, The effects of age and behavioral development on honey bee (Apis mellifera) flight performance. *J. Exp. Biol.* **212**, 2604–2611 (2009).

57. G. E. Robinson, R. E. Page, Z. Y. Huang, Temporal polyethism in social insects is a developmental process. *Anim. Behav.* **48**, 467–469 (1994).

58. Z.-Y. Huang, G. E. Robinson, Regulation of honey bee division of labor by colony age demography. *Behav. Ecol. Sociobiol.* **39**, 147–158 (1996).

59. I. Leoncini, Y. Le Conte, G. Costagliola, E. Plettner, A. L. Toth, M. Wang, Z. Huang, J.-M. Bécard, D. Crauser, K. N. Slessor, G. E. Robinson, Regulation of behavioral maturation by a primer pheromone produced by adult worker honey bees. *Proc. Natl. Acad. Sci. U.S.A.* **101**, 17559–17564 (2004).

60. A. C. Geffre, T. Gernat, G. P. Harwood, B. M. Jones, D. M. Gysi, A. R. Hamilton, B. C. Bonning, A. L. Toth, G. E. Robinson, A. G. Dolezal, Honey bee virus causes context-dependent changes in host social behavior. *Proc. Natl. Acad. Sci. U.S.A.* **117**, 10406–10413 (2020).

61. T. Durand, A. Bonjour-Dalmon, E. Dubois, Viral co-infections and antiviral immunity in honey bees. *Viruses* **15**, 1217 (2023).

62. V. Doublet, M. A. Y. Oddie, F. Mondet, E. Forsgren, B. Dahle, E. Furuseth-Hansen, G. R. Williams, L. De Smet, M. E. Natsopoulou, T. E. Murray, E. Semberg, O. Yañez, D. C. de Graaf, Y. Le Conte, P. Neumann, E. Rimstad, R. J. Paxton, J. R. de Miranda, Shift in virus composition in honeybees (Apis mellifera) following worldwide invasion by the parasitic mite and virus vectorVarroa destructor. *R. Soc. Open Sci.* **11**, 231529 (2024).

63. L. Bailey, The multiplication and spread of sacbrood virus of bees. *Ann. Appl. Biol.* **63**, 483–491 (1969).

64. J. Li, T. Wang, J. D. Evans, R. Rose, Y. Zhao, Z. Li, J. Li, S. Huang, M. Heerman, C. Rodríguez-García, O. Banmeke, J. R. Brister, E. L. Hatcher, L. Cao, M. Hamilton, Y. Chen, The phylogeny and pathogenesis of sacbrood virus (SBV) infection in European honey bees, *Apis mellifera*. *Viruses* **11**, 61 (2019).

65. B. Heinrich, How the honey bee regulates its body temperature. *Bee World* **77**, 130–137 (1996).

66. H. Bernd, Thermoregulation of African and European Honeybees during Foraging, Attack, and Hive Exits and Returns. *J. Exp. Biol.* **80**, 217–229 (1979).

67. A. Dalmon, M. Peruzzi, Y. L. Conte, C. Alaux, M. Pioz, Temperature-driven changes in viral loads in the honey bee Apis mellifera. *J. Invertebr. Pathol.* **160**, 87–94 (2019).

68. C. Bordier, H. Dechatre, S. Suchail, M. Peruzzi, S. Soubeyrand, M. Pioz, M. Pelissier, D. Crauser, Y. L. Conte, C. Alaux, Colony adaptive response to simulated heat waves and consequences at the individual level in honeybees (Apis mellifera). *Sci. Rep.* **7**, 3760 (2017).

69. S. A. Adamo, C. E. Linn, R. R. Hoy, The role of neurohormonal octopamine during 'fight or flight' behaviour in the field cricket Gryllus bimaculatus. *J. Exp. Biol.* **198**, 1691–1700 (1995).

70. Y. Li, J. Hoffmann, Y. Li, F. Stephano, I. Bruchhaus, C. Fink, T. Roeder, Octopamine controls starvation resistance, life span and metabolic traits in Drosophila. *Sci. Rep.* **6**, 35359 (2016).

71. V. Corby-Harris, M. E. Deeter, L. Snyder, C. Meador, A. C. Welchert, A. Hoffman, B. T. Obernesser, Octopamine mobilizes lipids from honey bee (Apis mellifera) hypopharyngeal glands. *J. Exp. Biol.* **223**, jeb216135 (2020).

72. B. L. Fussnecker, B. H. Smith, J. A. Mustard, Octopamine and tyramine influence the behavioral profile of locomotor activity in the honey bee (Apis mellifera). *J. Insect Physiol.* **52**, 1083–1092 (2006).

73. R. Gmeinbauer, K. Crailsheim, Glucose utilization during flight of honeybee (Apis mellifera) workers, drones and queens, Drones and Queens. *J. Insect. Physiol.* **39**, 959–967 (1993).

74. G. A. Wright, S. W. Nicolson, S. Shafir, Nutritional physiology and ecology of honey bees. *Annu. Rev. Entomol.* **63**, 327–344 (2018).

75. A. G. Dolezal, J. Carrillo-Tripp, T. M. Judd, W. Allen Miller, B. C. Bonning, A. L. Toth, Interacting stressors matter: Diet quality and virus infection in honeybee health. *R. Soc. Open Sci.* **6**, 181803 (2019).

76. A.-M. Klein, B. E. Vaissière, J. H. Cane, I. Steffan-Dewenter, S. A. Cunningham, C. Kremen, T. Tscharntke, Importance of pollinators in changing landscapes for world crops. *Proc. R. Soc. B* **274**, 303–313 (2007).

77. N. W. Calderone, Insect pollinated crops, insect pollinators and US agriculture: Trend analysis of aggregate data for the period 1992-2009. *PLOS ONE* **7**, e37235 (2012).

78. S. D. Desai, R. W. Currie, Effects of wintering environment and parasite–pathogen interactions on honey bee colony loss in north temperate regions. *PLOS ONE* **11**, e0159615 (2016).

79. C. W. W. Pirk, J. R. de Miranda, M. Kramer, T. E. Murray, F. Nazzi, D. Shutler, J. J. M. van der Steen, C. van Dooremalen, Statistical guidelines for Apis mellifera research. *J. Apicult. Res.* **52**, 24 (2013).

80. S. A. Alger, P. A. Burnham, A. K. Brody, Flowers as viral hot spots: Honey bees (Apis mellifera) unevenly deposit viruses across plant species. *PLOS ONE* **14**, e0221800 (2019).

81. T. Olgun, S. E. Everhart, T. Anderson, J. Wu-Smart, Comparative analysis of viruses in four bee species collected from agricultural, urban, and natural landscapes. *PLOS ONE* **15**, e0234431 (2020).

82. K. F. Daughenbaugh, I. Kahnonitch, C. C. Carey, A. J. McMenamin, T. Wiegand, T. Erez, N. Arkin, B. Ross, B. Wiedenheft, A. Sadeh, N. Chejanovsky, Y. Mandelik, M. L. Flenniken,

Metatranscriptome analysis of sympatric bee species identifies bee virus variants and a new virus, andrena-associated bee virus-1. *Viruses* **13**, 291 (2021).

83. E. A. Murray, J. Burand, N. Trikoz, J. Schnabel, H. Grab, B. N. Danforth, Viral transmission in honey bees and native bees, supported by a global black queen cell virus phylogeny. *Environ. Microbiol.* **21**, 972–983 (2019).

84. S. A. Alger, P. A. Burnham, H. F. Boncristiani, A. K. Brody, RNA virus spillover from managed honeybees (Apis mellifera) to wild bumblebees (Bombus spp.). *PLOS ONE* **14**, e0217822 (2019).

85. M. A. Furst, D. P. McMahon, J. L. Osborne, R. J. Paxton, M. J. Brown, Disease associations between honeybees and bumblebees as a threat to wild pollinators. *Nature* **506**, 364–366 (2014).

86. C. C. Grula, J. P. Rinehart, K. J. Greenlee, J. H. Bowsher, Body size allometry impacts flight-related morphology and metabolic rates in the solitary bee Megachile rotundata. *J. Insect Physiol.* **133**, 104275 (2021).

87. D. Bates, M. Machler, B. M. Bolker, S. C. Walker, Fitting linear mixed-effects models using lme4. *J. Stat. Softw.* **67**, 1–48 (2015).

88. A. M. Bolger, M. Lohse, B. Usadel, Trimmomatic: A flexible trimmer for Illumina sequence data. *Bioinformatics* **30**, 2114–2120 (2014).

89. B. J. Haas, A. Papanicolaou, M. Yassour, M. Grabherr, P. D. Blood, J. Bowden, M. B. Couger, D. Eccles, B. Li, M. Lieber, M. D. MacManes, M. Ott, J. Orvis, N. Pochet, F. Strozzi, N. Weeks, R. Westerman, T. William, C. N. Dewey, R. Henschel, R. D. LeDuc, N. Friedman, A. Regev, De novo transcript sequence reconstruction from RNA-seq using the Trinity platform for reference generation and analysis. *Nat. Protoc.* **8**, 1494–1512 (2013).

90. M. G. Grabherr, B. J. Haas, M. Yassour, J. Z. Levin, D. A. Thompson, I. Amit, X. Adiconis, L. Fan, R. Raychowdhury, Q. Zeng, Z. Chen, E. Mauceli, N. Hacohen, A. Gnirke, N. Rhind, F. di Palma, B. W. Birren, C. Nusbaum, K. Lindblad-Toh, N. Friedman, A. Regev, Full-length

transcriptome assembly from RNA-seq data without a reference genome. *Nat. Biotechnol.* **29**, 644–652 (2011).

91. S. F. Altschul, T. L. Madden, A. A. Schäffer, J. Zhang, Z. Zhang, W. Miller, D. J. Lipman, Gapped BLAST and PSI-BLAST: A new generation of protein database search programs. *Nucleic Acids Res.* **25**, 3389–3402 (1997).
